# LncRNA DSCAM-AS1 interacts with YBX1 to promote cancer progression by forming a positive feedback loop that activates FOXA1 transcription network

**DOI:** 10.7150/thno.47830

**Published:** 2020-08-29

**Authors:** Yin Zhang, Yong-Xin Huang, Dan-Lan Wang, Bing Yang, Hai-Yan Yan, Le-Hang Lin, Yun Li, Jie Chen, Li-Min Xie, Yong-Sheng Huang, Jian-You Liao, Kai-Shun Hu, Jie-Hua He, Phei Er Saw, Xiaoding Xu, Dong Yin

**Affiliations:** 1 Guangdong Provincial Key Laboratory of Malignant Tumor Epigenetics and Gene Regulation, Research Center of Medicine, Sun Yat-sen Memorial Hospital, Sun Yat-sen University, Guangzhou 510120, P. R. China.; 2 Medical Research Center, Sun Yat-sen Memorial Hospital, Sun Yat-sen University, Guangzhou 510120, P. R. China.

**Keywords:** FOXA1, lncRNAs, super-enhancer, lung adenocarcinoma, breast cancer, ERα

## Abstract

**Rationale:** The forkhead box A1 (FOXA1) is a crucial transcription factor in initiation and development of breast, lung and prostate cancer. Previous studies about the FOXA1 transcriptional network were mainly focused on protein-coding genes. Its regulatory network of long non-coding RNAs (lncRNAs) and their role in FOXA1 oncogenic activity remains unknown.

**Methods:** The Cancer Genome Atlas (TCGA) data, RNA-seq and ChIP-seq data were used to analyze FOXA1 regulated lncRNAs. RT-qPCR was used to detect the expression of DSCAM-AS1, RT-qPCR and Western blotting were used to determine the expression of FOXA1, estrogen receptor α (ERα) and Y box binding protein 1 (YBX1). RNA pull-down and RIP-qPCR were employed to investigate the interaction between DSCAM-AS1 and YBX1. The effect of DSCAM-AS1 on malignant phenotypes was examined through *in vitro* and *in vivo* assays.

**Results:** In this study, we conducted a global analysis of FOXA1 regulated lncRNAs. For detailed analysis, we chose lncRNA DSCAM-AS1, which is specifically expressed in lung adenocarcinoma, breast and prostate cancer. The expression level of DSCAM-AS1 is regulated by two super-enhancers (SEs) driven by FOXA1. High expression levels of DSCAM-AS1 was associated with poor prognosis. Knockout experiments showed DSCAM-AS1 was essential for the growth of xenograft tumors. Moreover, we demonstrated DSCAM-AS1 can regulate the expression of the master transcriptional factor FOXA1. In breast cancer, DSCAM-AS1 was also found to regulate ERα. Mechanistically, DSCAM-AS1 interacts with YBX1 and influences the recruitment of YBX1 in the promoter regions of FOXA1 and ERα.

**Conclusion:** Our study demonstrated that lncRNA DSCAM-AS1 was transcriptionally activated by super-enhancers driven by FOXA1 and exhibited lineage-specific expression pattern. DSCAM-AS1 can promote cancer progression by interacting with YBX1 and regulating expression of FOXA1 and ERα.

## Introduction

The forkhead box A1 (FOXA1) protein is a member of a group of special transcription factors (TF) called pioneer factors. These pioneer factors bind to condensed, inactive chromatin and initiate chromatin remodeling, which results in the accessibility of other transcription factors in this region 1. FOXA1 binds to its co-factors to transcriptionally activates target genes. FOXA1 plays a crucial role in tumorigenesis of breast, prostate and lung cancers 2-5. In prostate cancer, FOXA1 is indispensable in androgen receptor (AR)-mediated gene regulation by interacting directly with AR and co-occupying chromatin. FOXA1 mutation is one of the most prevalent genomic alteration in prostate cancer, appearing in 11% of cases 6. FOXA1 can drive the transformation of normal prostate epithelial cells combining with HOXB13 7. In breast cancer, FOXA1 interacts with estrogen receptor α (ERα), which is necessary for activating the expression of downstream oncogenes that promote tumor malignancy 8. Moreover, FOXA1 is required not only for maintaining luminal-specific gene expression, but also for suppressing genes specific for basal breast cancer cells 9. Even more interestingly, FOXA1 binding events are independent of hormonal signaling, which suggests FOXA1 may function as oncogene independent of hormonal receptors 10. In non-small-cell lung cancer (NSCLC) cells, FOXA1 plays oncogenic roles in cell growth and epithelial to mesenchymal transition (EMT) 11, 12. FOXA1 is also reported to participate in the suppression of squamous identity in lung cancer 13.

Long non-coding RNAs (lncRNAs) refer to non-coding RNAs consisting of >200 nucleotides. Increasing studies have reported lncRNAs play vital roles in cancer cell proliferation, metastasis and chemoresistance 14-17. Moreover, the lncRNAs tend to exhibit higher tissue-specific expressions than protein coding genes 18-20. Although the expression specificity of lncRNAs provides opportunities to explore new biomarkers and drug targets 21, how to identify lncRNA involved in key regulatory networks is still challenging.

For the importance of FOXA1 in tumorigenesis, many studies have been conducted to explore its transcription landscape. However, these studies were mainly focused on protein-coding genes. Uncovering its regulatory network, especially at the lncRNA layer, will be beneficial towards understanding its pathogenesis as well as the prevention and treatment of cancer.

In this study, we conducted a systemic analysis of FOXA1-regulated oncogenic lncRNAs. We chose a FOXA1 inducible lncRNA DSCAM-AS1 that expressed in lung adenocarcinoma, prostate and breast cancer specifically. DSCAM-AS1 has been reported to be over-expressed in a fraction of breast cancer and lung cancer cases in a lineage-dependent way 22-25. Our study revealed that the lineage-specific expression of DSCAM-AS1 was regulated by the FOXA1-driven super-enhancers (SEs) and DSCAM-AS1 can regulate expression of FOXA1 and ERα to maintain the characteristics of indicated cancer cells. Mechanistically, DSCAM-AS1 was found to interact with Y box binding protein 1 (YBX1) and influence the recruitment of YBX1 in the promoter regions of FOXA1 and ERα.

## Materials and Methods

### Datasets and computational analysis

The lncRNA expression data of Pan-cancer were downloaded from MiTranscriptome 26 (http://www.mitranscriptome.com/). The mRNA expression of TCGA data were downloaded from Broad Dashboard-Stddata (https://confluence.broadinstitute.org/display/GDAC/Dashboard-Stddata).

The ChIP-seq data were downloaded from ENCODE portal 27 (https://www.encodeproject.org/) and Cistrome 28. The ChIP-seq data of lung adenocarcinoma (LUAD) cells were downloaded from DBTSS portal 29 (https://dbtss.hgc.jp/). The Hi-C data were downloaded from ENCODE portal. The CNVs' status was analyzed by using the cBioPortal database (https://www.cbioportal.org/). All datasets used in this study are listed in Table S4. The gene expression in cell lines were obtained from the Cancer Cell Line Encyclopedia (CCLE).

Reads of ChIP-seq were aligned to the human reference genome (Hg38) using BOWTIE (version 1.1.2, with parameter v 1, m 10). The peaks were generated by MACS (version 14, with default parameters) 30, the super enhancers were analyzed using ROSE 31 with default parameters. The FOXA1 peaks within ±10 kb region of transcriptional start sites (TSS) of lncRNAs (GENCODE.v23) were considered as potential FOXA1 binding sites. Si-FOXA1 RNA-seq data were downloaded from GEO database (GSE83785) 32 and analyzed by RSEM 33 and edgeR 34 using default parameters.

### Cell lines, cell culture and treatment

Human breast cancer cell lines MCF7 and T47D, human lung adenocarcinoma cell lines NCI-H1573 and NCI-H1437, prostate cancer cell line 22Rv1, and lentivirus packaging HEK293T cell were obtained from the American Type Culture Collection. MCF7 and HEK293T cell lines were maintained in Dulbecco's Modified Eagle Medium (DMEM, Gibco, #11995065) containing 10% fetal bovine serum (Gibco, #10099141) and 1% penicillin-streptomycin (Beyotime, #C0222). T47D and 22Rv1 cell lines were cultured in Roswell Park Memorial Institute (RPMI) 1640 medium (Gibco, #11875500) supplied with 10% fetal bovine serum and 1% penicillin-streptomycin. NCI-H1437 and NCI-H1573 cell lines were propagated in RPMI-1640 supplemented with 10% fetal bovine serum, 1% penicillin-streptomycin, 1% GlutaMAX (Gibco, #35050061), 1% Non-Essential Amino Acids (Gibco, #11140050) and 1% Sodium Pyruvate (Gibco, #11360070). All cell lines were grown at 37°C in a humidified atmosphere of 5% CO_2_. MCF7 cells were treated with a series concentration of JQ-1(+) for 8 hours followed by RNA extraction and RT-qPCR to determine the expression of DSCAM-AS1.

### RNAi and cell transfection

DSCAM-AS1, FOXA1 and YBX1 in MCF7, T47D, NCI-H1437 and NCI-H1573 cell lines were knocked down by small interfering RNAs (siRNAs). siRNAs were synthesized by GenePharma Co. (Shanghai, China). The sequences of siRNA are listed in Table S3. siRNAs were transfected at a final concentration of 50 nM using Lipofectamine™ RNAiMAX Transfection Reagent (Invitrogen, #13778150) with Opti-MEM (Gibco, #31985070) using reverse transfection protocol according to the manufacturer's instructions. Then the RNAs were harvested at 48 hours and proteins were harvested at 72 hours after siRNA transfection.

### Lentiviral CRISPR/Cas9 plasmid construction and cell infection

For stable knockout of DSCAM-AS1, MCF7 and NCI-H1437 cells were treated with lentiviral CRISPR/Cas9 and a guide RNA system. We designed two gRNAs targeted to the upstream and downstream of DSCAM-AS1, which can result in two cleavage sites and deletion of DSCAM-AS1. The sequences of gRNAs are listed in Table S2. We cloned the target sequence into the lentiCRISPRv2 (Addgene #52961) backbone according to the manufacturer's instructions. For lentiviral production, a transfer plasmid was co-transfected into HEK293T cell with the packaging plasmids psPAX2 (Addgene #12260) and pMD2.G (Addgene #12259) using polyethylenimine transfection reagent. Supernatants containing viral particles were harvested at 24, 36, 48 and 60 hours after transfection, after which viral particles were concentrated by adding 44% PEG8000 (Sigma-Aldrich, #89510) and 4 M NaCl. The concentrated viruses were resuspended with PBS and stored in aliquots at -80°C. For lentiviral infection, indicated cells were infected with the lentiviruses in the presence of 2 mg/mL Polybrene (Sigma-Aldrich, St Louis, MO). Cells were infected by mixed viruses containing two gRNAs targeted to the upstream and downstream of DSCAM-AS1 locus.

### Chromatin Immunoprecipitation-qPCR Analysis

Chromatin immunoprecipitation (ChIP) was performed as described previously 35. Cells were fixed with 1% formaldehyde, and nuclei were extracted. Chromatin/DNA complex was sheared in a sonicator. Sonicated lysates were cleared and incubated overnight at 4°C with magnetic beads coupled with one of the following antibodies: H3K27ac antibody (Abcam, ab4729), FOXA1 antibody (Abcam, ab23738), YBX1 antibody (Abcam, ab76149) or ERα antibody (Santa Cruz, sc-543). DNA was eluted and analyzed by qPCR. The ChIP-qPCR primers are listed in Table S1.

### Cell proliferation assays and cell cycle analysis

For MTT (3-(4, 5-dimethylthiazol-2-yl)-2, 5-diphenyl tetrazoliumbromide) assay, 2500 cells were plated in 96-well plates in 100 μl media containing 10% FBS, and cultured for 5 days. Cell viability was assessed at days 1, 3 and 5 using the MTT method. For colony formation assay, 1000 cells were seeded onto 6-well plates, and grown for 2 weeks. Colonies were fixed with 4% paraformaldehyde and stained with 0.1% crystal violet. The number of colonies were counted by Image J software. All assays were performed in triplicate. For cell cycle assay, 72 hours after siRNA transfection, cells were digested and washed with PBS and then fixed in 70% ethanol at 4°C overnight, then incubated with RNase and propidium iodide (PI) for 10 min and analyzed by flow cytometry.

### Xenograft in nude mice model

MCF7 cells (3 × 10^6^) stably expressing CRISPR/Cas9-NC, CRISPR/Cas9-gRNA1+3 were subcutaneously injected into the breast of eight 5-week-old female nude mice. A 0.72 mg 60-days estradiol pellet (Innovative Research of America, Sarasota, FL) was used for estrogen supplementation. Tumor volumes were measured (length × width^2^ × 0.5) and evaluated two or three times a week. After 18 days, mice were sacrificed and the tumors were excised. The animal study was approved by the Institutional Animal Care and Use Committee at the Sun Yat-sen University.

### RNA extraction and RT-qPCR

Total RNA was extracted by TRIzol Reagent (Invitrogen, #15596018), and 800 ng of isolated RNA were used for reverse transcription using PrimeScript™ RT reagent Kit with gDNA Eraser (Takara, #RR047A). The cDNA templates were analyzed by CFX96 qPCR System (Biorad). Expression of each gene was normalized to β-actin as internal reference, and quantified using the 2^-ΔΔ^ (ct) method. Gene specific primers were designed by Primer 3 software and synthesized by IGE Technologies. Primers are listed in Supplementary Table S1.

### Western blotting

Cells were lysed by RIPA buffer, supplemented with proteinase inhibitor cocktail (Bimake, #B14001) and phosphatase inhibitor cocktail (Bimake, #B15001). Protein quantification was determined by Bradford. Western blotting was performed by using SDS-PAGE gels and followed by protein transfer to 0.45 um PVDF membrane (Merck millipore), then incubated with 5% nonfat milk in PBST for blocking for 1h. Indicated antibodies were added to the surface of the membrane overnight at 4°C. Secondary antibodies (Transgen Biotech, #HS101) were incubated for 1 hour at room temperature. At last, membranes were visualized using ECL detection reagents (Beyotime, #P0018A).

### RNA pull down assays and mass spectrometry

RNA pull down assays were conducted using a tRNA scaffold to a Streptavidin aptamer (tRSA) system as previously reported 36. Briefly speaking, this method uses a Streptavidin RNA aptamer with a tRNA scaffold (tRSA) to achieve high affinity to Streptavidin beads. The lncRNA sequences were inserted downstream of tRSA and transcribed together with tRSA. The DSCAM-AS1 fragment (NCBI ID: AF401035) was cloned into a pcDNA3-tRSA vector using EcoRV and NotI restrict enzymes. The DSCAM-AS1 fragments were amplified from cDNA of MCF7 cells and the pcDNA3-tRSA plasmid were obtained from Addgene (#32200).

Cell lysates were prepared as follows: MCF7 and NCI-H1437 cells in a 100 mm dish were harvested and lysed by adding 1ml lysis buffer (10 mM HEPES pH 7.0, 200 mM NaCl, 1% Triton X-100, 10 mM MgCl_2_, 1 mM DTT with protease inhibitors and RNase inhibitor). Cell lysates were sonicated for 10 cycles of 30 seconds on, 30 seconds off (Bioruptor Plus, Diagenode), then incubated on ice for another 20 minutes, and centrifuged for 15 minutes at 12000 rpm, 4°C. Supernatants were transferred to new tubes and the amount of protein quantified by measuring the absorbance at 280 nm. For preclear, 200 μg per reaction protein was incubated with 30 μl washed Dynabeads MyOne Streptavidin C1 (Invitrogen, #65001) at 4°C for 4 hours. RNAs were synthesized using TranscriptAid T7 High Yield Transcription Kit (Thermofisher, #K0441). tRSA empty vector were used as negative control as previously reported 36, 37. 60 pmol of synthetic RNAs were denatured at 85°C for 5 minutes then cooled to room temperature with 10 mM HEPES and 10 mM MgCl_2_ for RNA folding. RNAs were applied to 30 μl washed streptavidin beads and incubated on a rotating shaker at 4°C for at least 20 minutes. All the precleared lysates were added to the RNA-beads and incubated for another 1.5 hours on a rotation shaker at 4°C. Beads were washed 5 times with lysis buffer. Captured proteins were heated for denature in the presence of 2X Laemmli Sample Buffer with β-ME (BIO-RAD) and separated by SDS-PAGE then analyzed by Western blotting or silver staining. The differential band indicated by silver staining was excised and used for mass spectrometry analysis.

### RNA Immunoprecipitation Assay

RIP assay was performed as reported 38. Briefly, cells were treated with 0.3% formaldehyde and the cross-linked reaction was stopped by adding 0.125 M glycine. Cells were resuspended in 1 ml of RIPA buffer, YBX1 antibody (Abcam, #ab76149) or IgG (Invitrogen, #SA5-10197) with Dynabeads proteinG (Invitrogen, #10004D) were added and incubated overnight at 4ºC. Samples were then washed and treated with proteinase K. RNA samples were purified by phenol chloroform extraction, followed by RT-qPCR to measure DSCAM-AS1 transcripts enrichment. Proteins isolated before proteinase K treatment from the beads were detected by Western blotting analysis.

### Statistical analysis

All data were shown as mean ± standard deviation (SD) processed by GraphPad Prism 7.0. Wilcoxon rank-sum test was used for comparing expression of genes and lncRNAs in different groups of patients of TCGA data. Student's *t*-test was used to test for statistical significance of the differences between two different group parameters and One-Way ANOVA followed by Bonferroni test was used for multiple comparisons. *P* values <0.05 were considered statistically significant.

## Results

### DSCAM-AS1 is a lineage-specific oncogenic lncRNA regulated by FOXA1

**Figure 1:**
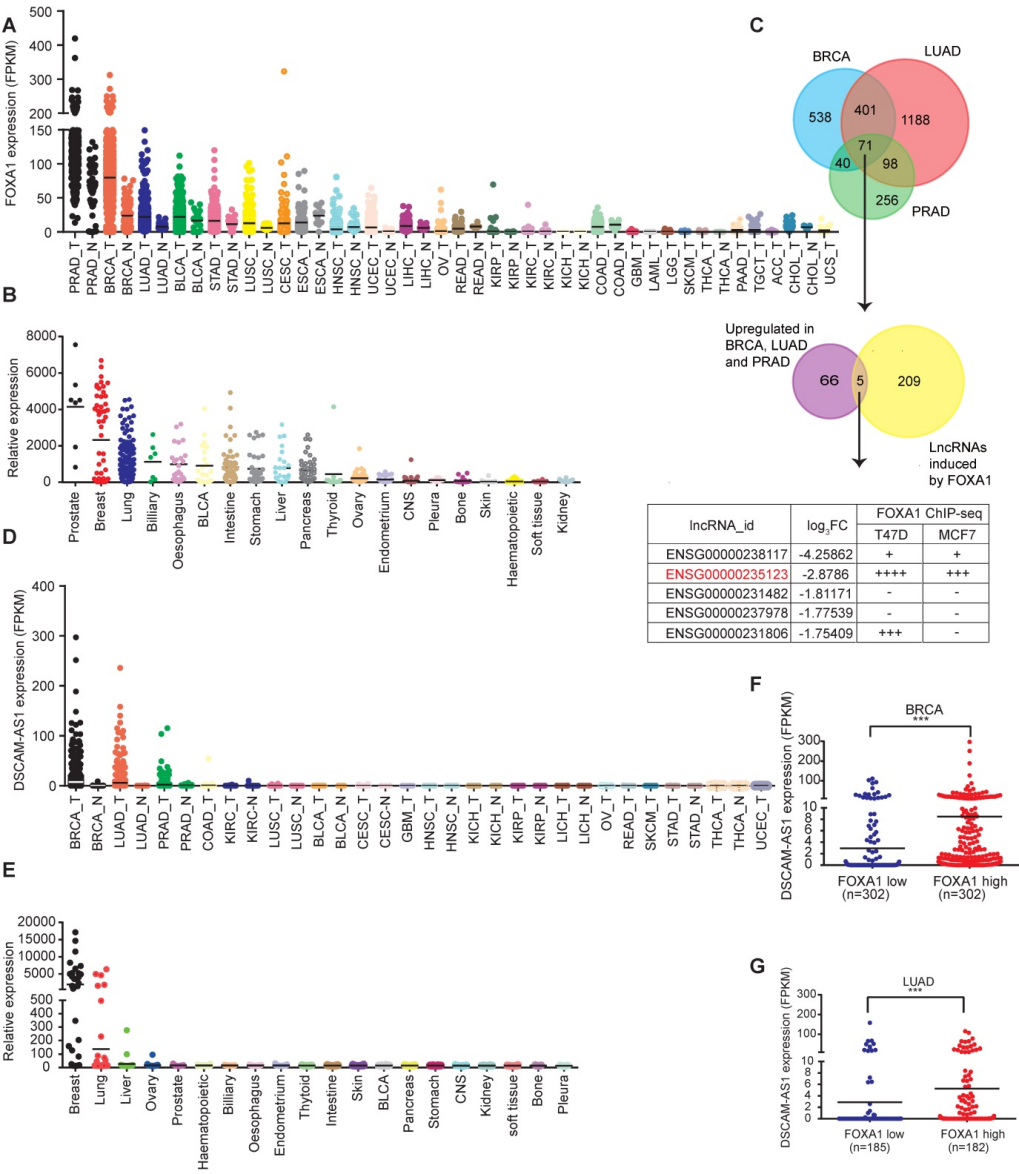
** Identification of lineage specific, FOXA1-regulated lncRNAs.** (**A**) Expression levels of FOXA1 in normal and tumor samples of The Cancer Genome Atlas. Each point represents one tissue sample. (**B**) Expression levels of FOXA1 in the Cancer Cell Line Encyclopedia project of cancer cell lines. Each point represents one cell line. (**C**) Venn diagram showing the screening of FOXA1-specific lncRNAs. Aberrantly overexpressed lncRNAs were analyzed in BRCA, PRAD and LUAD using the TCGA database. A total of 71 lncRNAs were highly expressed in these three cancer types (fold change [FC] ≥3, *P* <0.05). These lncRNAs were then overlapped with 214 FOXA1 induced lncRNAs in the T47D cell line (FC≥ 2, *P* <0.05) identified by RNA-seq. Five lncRNAs intersections were then analyzed for potential association in the promoter region with FOXA1 using ChIP-seq data in ENCODE datasets. (**D**) Expression levels of DSCAM-AS1 in The Atlas of Noncoding RNAs in Cancer project of normal and tumor samples. Each point represents one tissue sample. (**E**) Expression levels of DSCAM-AS1 in cancer cell lines of the Cancer Cell Line Encyclopedia project. Each point represents one cell line. (**F-G**) The correlation between FOXA1 and DSCAM-AS1 expression levels in BRCA (F) and LUAD (G) samples from TANRIC dataset.

To reveal the pivotal oncogenic role of FOXA1 regulatory network, we conducted integrated analysis of FOXA1-responsive lncRNAs with oncogenic functions. We first investigated the expression pattern of FOXA1 in TCGA pan-cancer data. We found prostate adenocarcinoma (PRAD), breast cancer (BRCA) and lung adenocarcinoma (LUAD) showed the most significantly elevated expression of FOXA1 (Figure 1A, Figure S1A). Similar results were observed in the Cancer Cell Line Encyclopedia (CCLE) dataset (Figure 1B). These results were in accordance with previous reporting that FOXA function as a oncogene in PRAD, LUAD and BRCA tumors 6, 11, 39. So, we next analyzed aberrantly expressed lncRNAs in PRAD, BRCA and LUAD. We found that 71 lncRNAs were over-expressed in these three cancer types (Figure 1C, Table S5). By analyzing public si-FOXA1 RNA-seq data reported by Nacht *et al.* 32 (GSE83785), we identified 214 lncRNAs that were significantly reduced after silencing of FOXA1 in T47D cells. From these data, five overlapping lncRNAs were selected for analyzing potential FOXA1 binding sites in the promoter regions using public ChIP-seq data (Table S4). Of them, DSCAM-AS1 (ENSG00000235123) was found to have the largest number of potential binding sites around TSS (± 10kb) (Figure 1C). Therefore, DSCAM-AS1 was selected for further investigation.

To characterize the expression of DSCAM-AS1, its expression levels were analyzed in TCGA data, showing it was specifically expressed in breast cancer, lung cancer and prostate cancer but not in adjacent normal tissues (Figure 1D). This unique expression pattern was also observed in the CCLE dataset (Figure 1E). In lung cancer, about 1/5 (93 of 488) LUAD samples showed high expression of DSCAM-AS1 (FPKM>2), but the expression level in lung squamous cell carcinoma (LUSC) was very low (only 2 of 230 patients FPKM>2, Figure S1B). In breast cancer, it was reported that DSCAM-AS1 showed much higher expression in ER^+^ compared to ER^-^ breast cancer tissues 22, 23. In spite of high expression levels of DSCAM-AS1 in some prostate cancer patients, its expression level is very low in the prostate cancer cell line (Figure S1C-D), which could be due to a limited number of human prostate cancer cell lines 40. So, we focused our study on ER^+^ breast cancer and lung adenocarcinoma and cell lines with high expressions of DSCAM-AS1 (Figure S1C-D).

### DSCAM-AS1 was a super-enhancer-driven lncRNA

**Figure 2:**
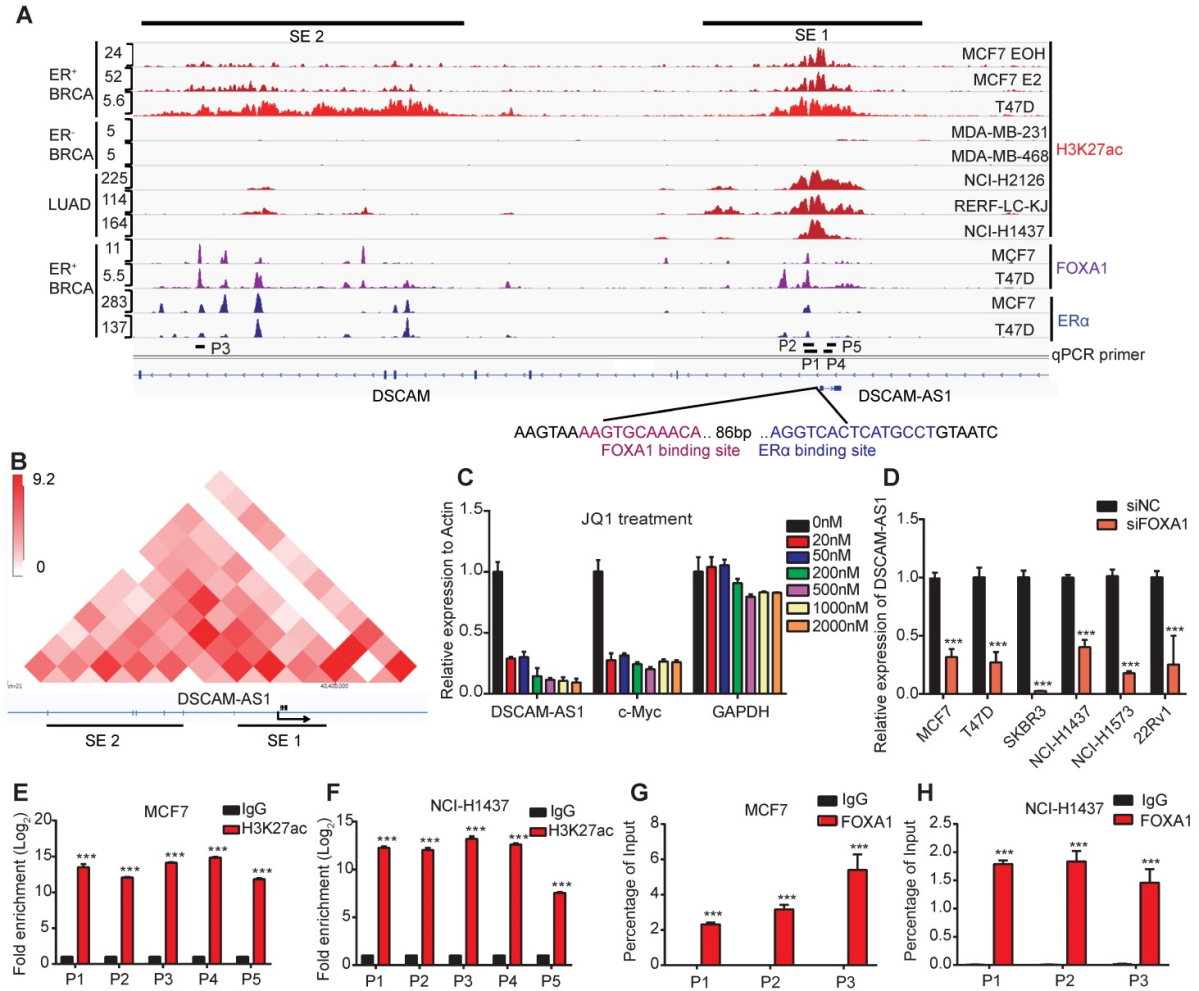
** DSCAM-AS1 is positively and directly regulated by FOXA1 driven super enhancers.** (**A**) ChIP-seq profiles of H3K27ac, FOXA1 and ERα at representative super-enhancer-associated gene loci in lung adenocarcinoma and breast cancer cell lines. The predicted SEs are depicted as black bars. The y-axis represents reads per million (rpm) of ChIP-seq. The location of ChIP-qPCR primers is indicated with a short black line. The FOXA1 and ERα binding motif are shown on the bottom. (**B**) The heat map of Hi-C interactions generated using 3D Genome Browser (http://promoter.bx.psu.edu/hi-c/index.html). The SEs and transcriptional directions are indicated below the heat map. (**C**) The expression levels of DSCAM-AS1, c-Myc and GAPDH were analyzed by RT-qPCR after treating with different concentrations of JQ1 in MCF7 cells. (**D**) Indicated cell lines of BRCA, LUAD and PRAD were transfected with siNC and siFOXA1 pool, the expression level of DSCAM-AS1 were detected by RT-qPCR. (**E-F**) ChIP-qPCR of H3K27ac using different primers in MCF7 (E) and NCI-H1437 (F) cell lines, the locations of primers were indicated in (A). (**G-H**) ChIP-qPCR showed enrichment of FOXA1 in MCF7 (G) and NCI-H1437 cells (H). The position of qPCR is indicated in (A).

Since the aberrant expression of genes in cancers is usually caused by copy number variations (CNVs), epigenetic and transcriptional dysregulations, we firstly analyzed the CNVs status at the gene body of DSCAM-AS1. Surprisingly, no significant copy number amplification was observed in neither breast cancer nor lung adenocarcinoma (Figure S1E), indicating CNVs are not the reason for overexpression of DSCAM-AS1. Super-enhancers are reported to be frequently involved in controlling lineage-specific expression and defining cell identity 41, we hypothesized that the unique expression pattern of DSCAM-AS1 may be regulated by super-enhancer. The H3K27ac is well recognized as a marker for active enhancers and super-enhancer 42, therefore, we analyzed the H3K27ac signals in breast and lung cancer cell lines. DSCAM-AS1 is an antisense lncRNA located in a large intron (about 320 kb) of the DSCAM gene. Recently, the location of DSCAM-AS1 have been identified to have two super-enhancer signals 25. Using H3k27ac ChIP-seq data, two super-enhancer clusters were confirmed to exist in ER^+^ but were absent in ER^-^ breast cancer cell lines (Figure 2A). One super-enhancer (SE1) was found to cover the entire gene body of DSCAM-AS1, including the promoter region (Figure 2A). Another super-enhancer cluster (SE2) was about 60kb upstream of DSCAM-AS1. We also confirmed that there was a significant ERα binding peak in the promoter region of DSCAM-AS1, which had been reported previously 22, 23. To further characterize the interaction between SEs and DSCAM-AS1, we analyzed Hi-C data of the T47D cell line. The interactions between SE2 with SE1 as well as DSCAM-AS1 TSS were observed by Hi-C heat map (Figure 2B). Super-enhancer-associated genes are highly sensitive to transcriptional inhibitors, such as the CDK7 inhibitor THZ1 and BRD4 inhibitor JQ1, even at low concentrations 43. THZ1 was reported mainly to play a role in triple negative but not hormone receptor-positive breast cancer cells 44, so we used different concentrations of JQ1 to explore the effect on the expression of DSCAM-AS1. As shown in Figure 2C, like the well-documented super-enhancer-driven gene c-Myc, a very low concentration (20 nM) of JQ1 resulted in the significant reduction of DSCAM-AS1, while the expression of the housekeeping gene GAPDH and host gene DSCAM (Figure S1F) were almost unchanged. Moreover, ChIP-qPCR assay showed a significant enrichment of H3K27ac in both upstream and gene body regions of DSCAM-AS1 (Figure 2E-F). Taken together, these data revealed DSCAM-AS1 was transcriptionally regulated by super-enhancers.

### DSCAM-AS1 is transcriptionally activated by FOXA1

Super-enhancers are large clusters of enhancers with aberrantly high levels of TFs binding to drive transcription of genes 45. To find potential TFs that activate expression of DSCAM-AS1, we conducted a motif analysis of the promoter region using ChIPBase 46 and JASPAR 47. FOXA1 and ERα were among the top candidates (Figure S1G-H), with the binding motif near the TSS. Moreover, ChIP-seq data showed significant peaks of FOXA1 near the TSS, which contains conical FOXA1 binding motifs (Figure 2A). We analyzed the expression patterns between FOXA1 and DSCAM-AS1 using TCGA breast and lung cancer datasets. In FOXA1-high tumor samples, a significantly higher expression of DSCAM-AS1 was found in both breast cancer and lung adenocarcinoma patients (Figure 1F-G). FOXA1 has been reported as a pioneer TF and serves as a key determinant of estrogen receptor transcriptional functions 8, 48. To further verify the occupation of FOXA1 in the promoter region of DSCAM-AS1, ChIP-qPCR assay was performed on potential FOXA1 and ERα binding sites identified by ChIP-seq. A significant enrichment of FOXA1 in MCF7 and NCI-H1437 was detected (Figure 2G-H). To further verify the regulation roles of FOXA1 on DSCAM-AS1, we knocked down FOXA1 by siRNAs in lung adenocarcinoma, breast cancer, and prostate cancer cell lines, respectively, qPCR and Western blotting showed these siRNAs could effectively silence FOXA1 levels. A dramatic reduction of DSCAM-AS1 levels was observed after FOXA1 silencing (Figure 2D, Figure S1J-Q). These data revealed that FOXA1 can directly bind to the promoter of DSCAM-AS1 and regulates its expression in lung adenocarcinoma, breast and prostate cancer cells. Taken together, DSCAM-AS1 is a direct target of FOXA1.

### Depletion of DSCAM-AS1 inhibits growth of breast cancer and lung adenocarcinoma cells

**Figure 3:**
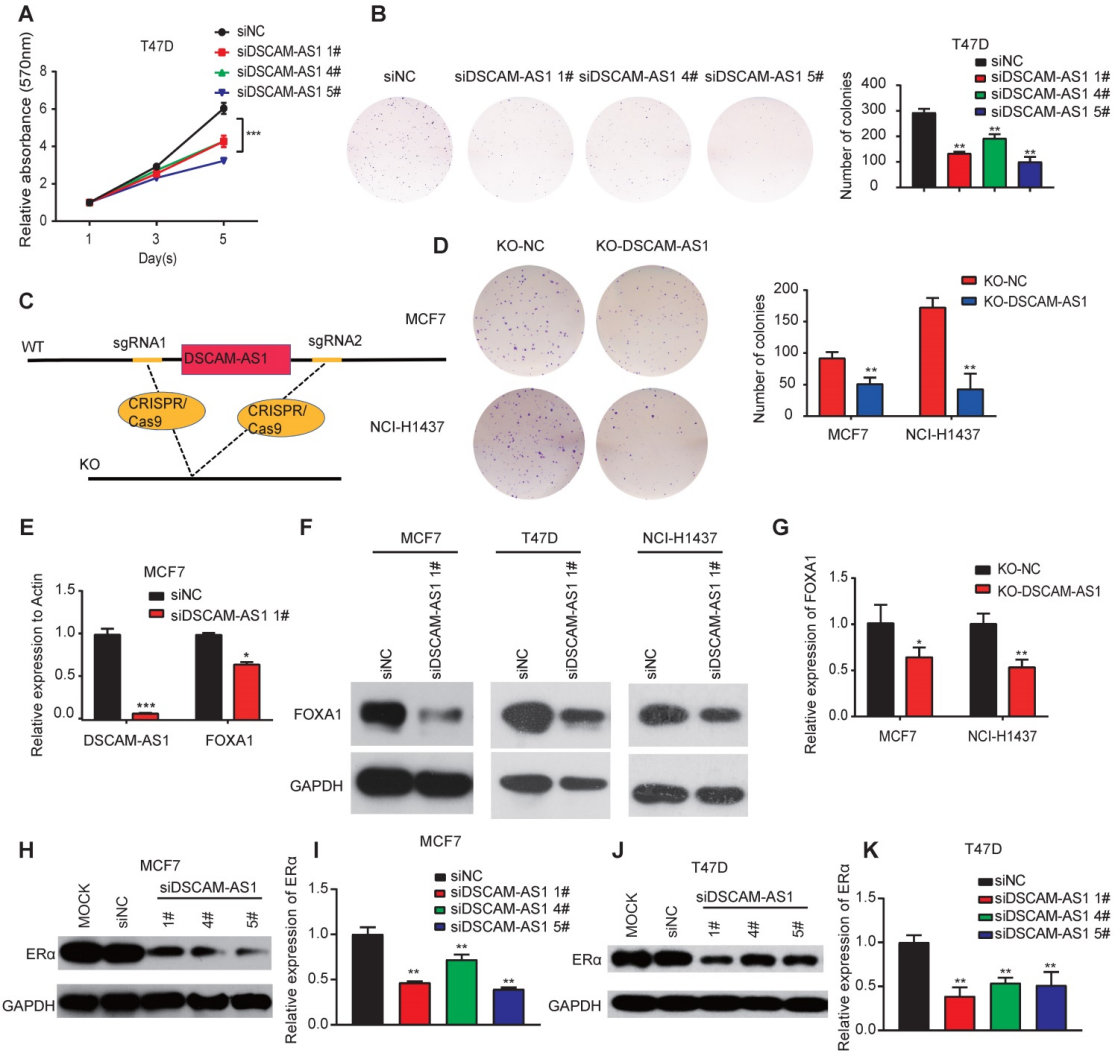
** DSCAM-AS1 plays oncogenic roles through regulation of FOXA1 and ERα expression.** (**A, B**) MTT (A) and colony formation assay (B) showed a significant reduction of cell growth after knocking down of DSCAM-AS1 with three individual siRNAs respectively. (**C**) Schematic diagram showed knocking out of DSCAM-AS1 using CRISPR/Cas9. (**D**) Decrease of colony formation after knocking out of DSCAM-AS1 in MCF7 and NCI-H1437 cells. (**E-F**) The mRNA (E) and protein (F) levels of FOXA1 after silencing of DSCAM-AS1. (**G**) qPCR showing the mRNA level of FOXA1 after knocking out of DSCAM-AS1 in MCF7 and NCI-H1437 cells. (**H-K**) Protein level of ERα after silencing DSCAM-AS1 using siRNAs in MCF7 (H) and T47D (J) cells. The mRNA level of ERα in MCF7 (I) and T47D (K) is also shown.

We further investigated the biological roles of DSCAM-AS1 in breast cancer and lung adenocarcinoma cells. We knocked down its expression by three individual siRNAs. qPCR assay confirmed DSCAM-AS1 was successfully silenced (Figure S2C-D). We observed that silencing DSCAM-AS1 significantly reduced both the proliferation and colony growth of breast cancer cell lines (Figure 3A-B). Flow cytometry assay showed knock down of DSCAM-AS1 resulted in a dramatic inhibition of the G1-S transition in breast cancer cells (Figure S2A-B). Our result is consistent with previous work that DSCAM-AS1 play oncogenic roles in breast cancer 22, 23. Moreover, we also knocked out DSCAM-AS1 by CRISPR/Cas9 using two sgRNAs (Figure 3C). qPCR showed DSCAM-AS1 expressions were dramatically reduced, but the expression or splicing of host gene DSCAM was not affected (Figure S2E-F). Significant growth inhibition was observed in breast cancer and lung adenocarcinoma cells after knockout of DSCAM-AS1 (Figure 3D). We failed to build a homozygous DSCAM-AS1-deletion cells, which might be due to the fact that DSCAM-AS1 is vital for cancer cell growth.

### DSCAM-AS1 can regulate expression of FOXA1 and ERα

Super-enhancer-associated TFs often form positive feedback loops with each other, which are called core transcriptional regulatory circuitries (CRC). CRC are crucial for cell-type-specific transcriptional regulation in cells 49. To explore whether super-enhancer-associated lncRNA can also regulate the expression of TFs and be involved in forming CRC, we measured the expression of FOXA1 in breast cancer and lung adenocarcinoma cell lines after knockdown of DSCAM-AS1. qPCR assay showed the mRNA level of FOXA1 was also significantly reduced after silencing of DSCAM-AS1 (Figure 3E), which indicated DSCAM-AS1 may regulate FOXA1 expression at transcription level. Western blotting showed silencing of DSCAM-AS1 decreased FOXA1 at the protein level (Figure 3F). The decrease of FOXA1 expression was further confirmed by knocking out of DSCAM-AS1 using CRISPR/Cas9 in MCF7 and NCI-H1437 cells (Figure 3G).

ERα is a master regulator that co-binds with FOXA1 in ER^+^ breast cancer. This process is vital for the initiation and development of breast cancers 50-52. FOXA1 is also reported to be positively correlated with ERα 53, Niknafs* et al.* 22 and Minao *et al.* 23 found DSCAM-AS1 is regulated by ERα. So, we speculated DSCAM-AS1 may also regulate expression of ERα. As expected, ERα expression was remarkably reduced at both mRNA and protein levels after silencing or knockout of DSCAM-AS1 (Figure 3H-K, Figure S2G-H). These data showed DSCAM-AS1 can regulate expression of FOXA1 and ERα.

### DSCAM-AS1 interacts with YBX1

**Figure 4:**
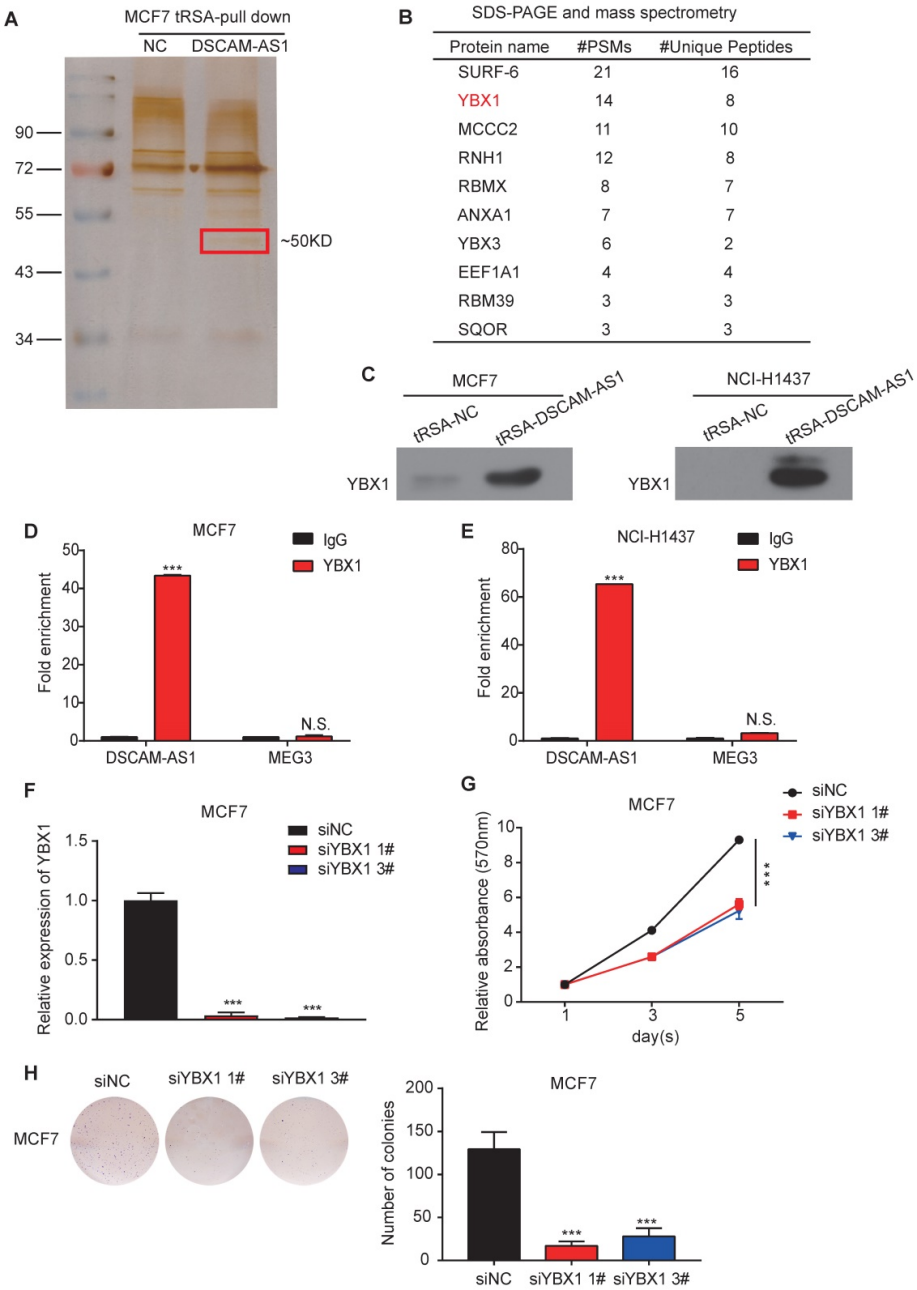
** DSCAM-AS1 interacts with YBX1.** (**A**) Silver staining of DSCAM-AS1-associated proteins after RNA pull-down using tRNA scaffold to a Streptavidin aptamer (tRSA) system; the empty tRSA vector is used as control. The band indicated in the red box is excised for mass spectrum analysis. (**B**) Mass spectrum results of the excised band. (**C**) Western blotting assay of proteins after RNA pull-down using YBX1 antibody. (**D-E**) RIP-qPCR showed enrichment of DSCAM-AS1 after immunoprecipitation of YBX1 in MCF7 (D) and NCI-H1437 (E). (**F**) qPCR showed the knocking down efficiency of YBX1 siRNAs. (**G-H**) MTT assay (G) and colony formation assay (H) showed the change of cell growth after knocking down YBX1.

To further explore the underlying molecular mechanism of the regulation of DSCAM-AS1 on FOXA1 and ERα, we conducted RNA pull-down assay in MCF7, where silver staining showed a significantly stronger band at ~50 KD compared to the control (Figure 4A). We further analyzed this band by mass spectrometry (Figure 4B). SURF-6 and YBX1 were among the top candidates obtained by mass spectrometry. Because the function of lncRNAs commonly rely on their interacting proteins 54, we first silenced SURF-6, but we failed to observe reduction of cell proliferation (Figure S3A-D). Therefore, another top-ranked protein, YBX1, attracted our interest. YBX1 is a DNA binding and RNA binding protein that plays important roles in regulating gene expression at both transcriptional and posttranscriptional levels. The oncogenic role of YBX1 in different kinds of tumors, including breast and lung cancer, have been also reported in recent years 55-57. The interaction between DSCAM-AS1 and YBX1 identified by RNA pull-down mass spectrometry was validated the by Western blotting using an anti-YBX1 antibody (Figure 4C). We further verified this interaction by RIP-qPCR assay, when compared to normal IgG, YBX1 resulted in a significant enrichment of DSCAM-AS1 in both MCF7 and NCI-H1437 cells (Figure 4D-E, Figure S4A-B). For the functional study, we knocked down YBX1 by siRNAs (Figure 4F), the proliferation of MCF7 cells was dramatically inhibited by using MTT and colony formation assay (Figure 4G-H). These data revealed that the function of DSCAM-AS1 relies on interaction with YBX1.

### Depletion of DSCAM-AS1 impairs YBX1 recruitment in promoter regions of FOXA1 and ERα

**Figure 5:**
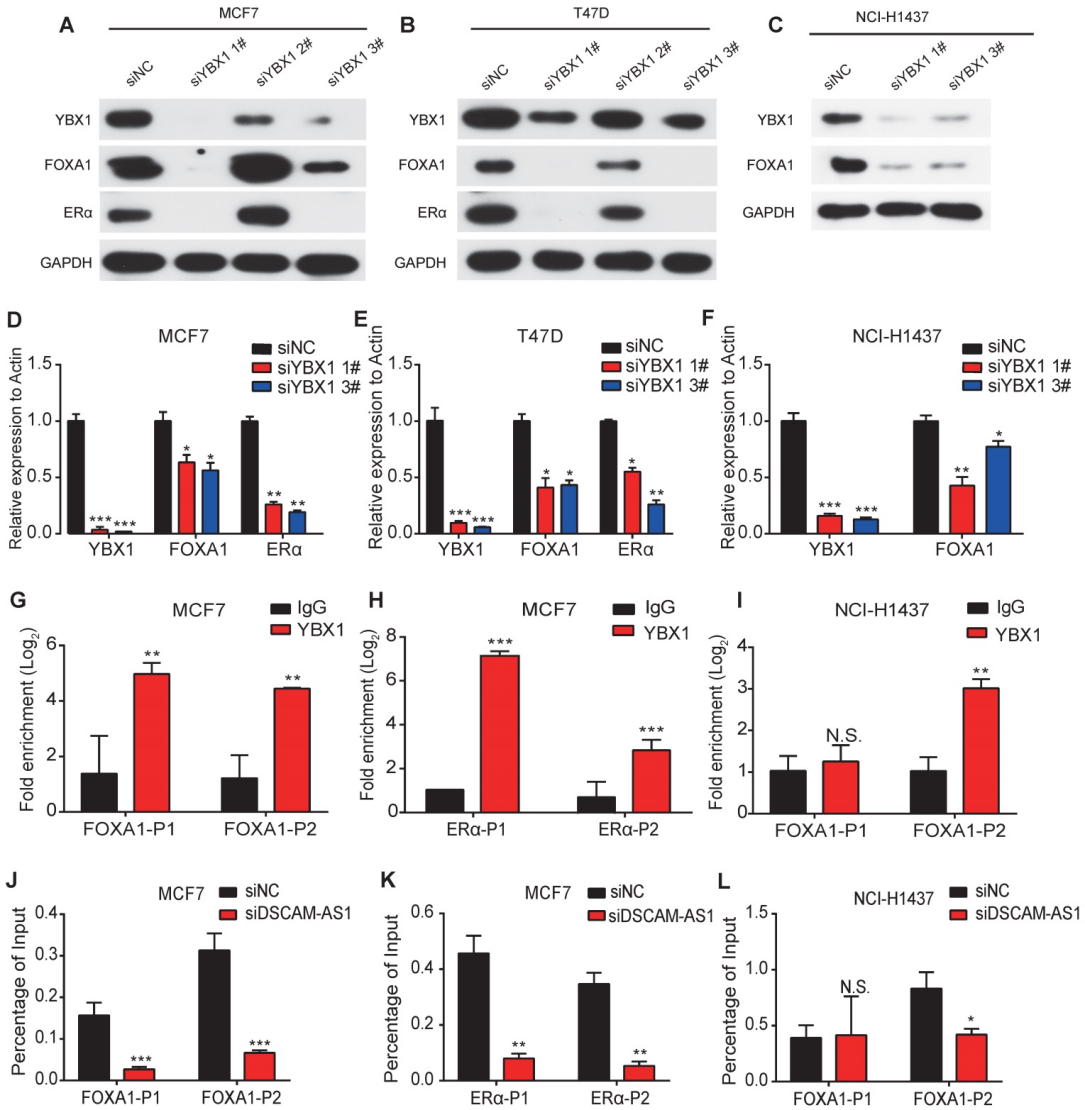
** DSCAM-AS1 enhances the recruitment of YBX1 in the promoter regions of FOXA1 and ERα to promote their expression.** (**A-C**) Western blotting showed the protein level of YBX1, FOXA1 and ERα after knocking down YBX1 in MCF7 (A), T47D (B) and NCI-H1437 (C). (**D-F**) qPCR showed the RNA level of YBX1, FOXA1 and ERα after knocking down YBX1 in MCF7 (D), T47D (E) and NCI-H1437 (F). (**G, I**) ChIP-qPCR showed enrichment of YBX1 at the promoter of FOXA1 in MCF7 (G) and NCI-H1437 (I) cell lines. (H) ChIP-qPCR showed enrichment of YBX1 at the promoter of ERα. (**J, L**) ChIP-qPCR results showed YBX1 recruitment change at the promoter of FOXA1 after silencing of DSCAM-AS1 in MCF7 (J) and NCI-H1437 (L) cell lines. (**K**) ChIP-qPCR indicated YBX1 recruitment change at the promoter of ERα after knocking down DSCAM-AS1 in NCI-H1437 cells.

In addition to its roles as an RNA binding protein, YBX1 also functions as a transcription factor 58. Previous studies showed lncRNA can bind to YBX1 and influence the transcriptional activities of YBX1 59. So, we speculated that DSCAM-AS1 may regulate expression of FOXA1 and ERα through YBX1. By analyzing the ChIP-seq data of YBX1, we observed significant peaks in the promoter region of FOXA1 and ERα (Figure S4C). These binding sites were further verified by ChIP-qPCR assay (Figure 5G-I). Furthermore, knock down of YBX1 resulted in dramatic suppression of FOXA1 and ERα at both mRNA and protein levels (Figure 5A-F), which revealed YBX1 could transcriptionally activate FOXA1 and ERα. Using ChIP-qPCR assay, we evaluated the change of occupation of YBX1 in the promoter region of FOXA1 and ERα. After silencing DSCAM-AS1, a significant reduction in recruitment of YBX1 was observed (Figure 5J-L). These data indicated depletion of DSCAM-AS1 impairs YBX1 binding in the promoter regions and reduces transcriptionally activate of FOXA1 and ERα.

### Clinical relevance and *in vivo* function study of DSCAM-AS1

**Figure 6:**
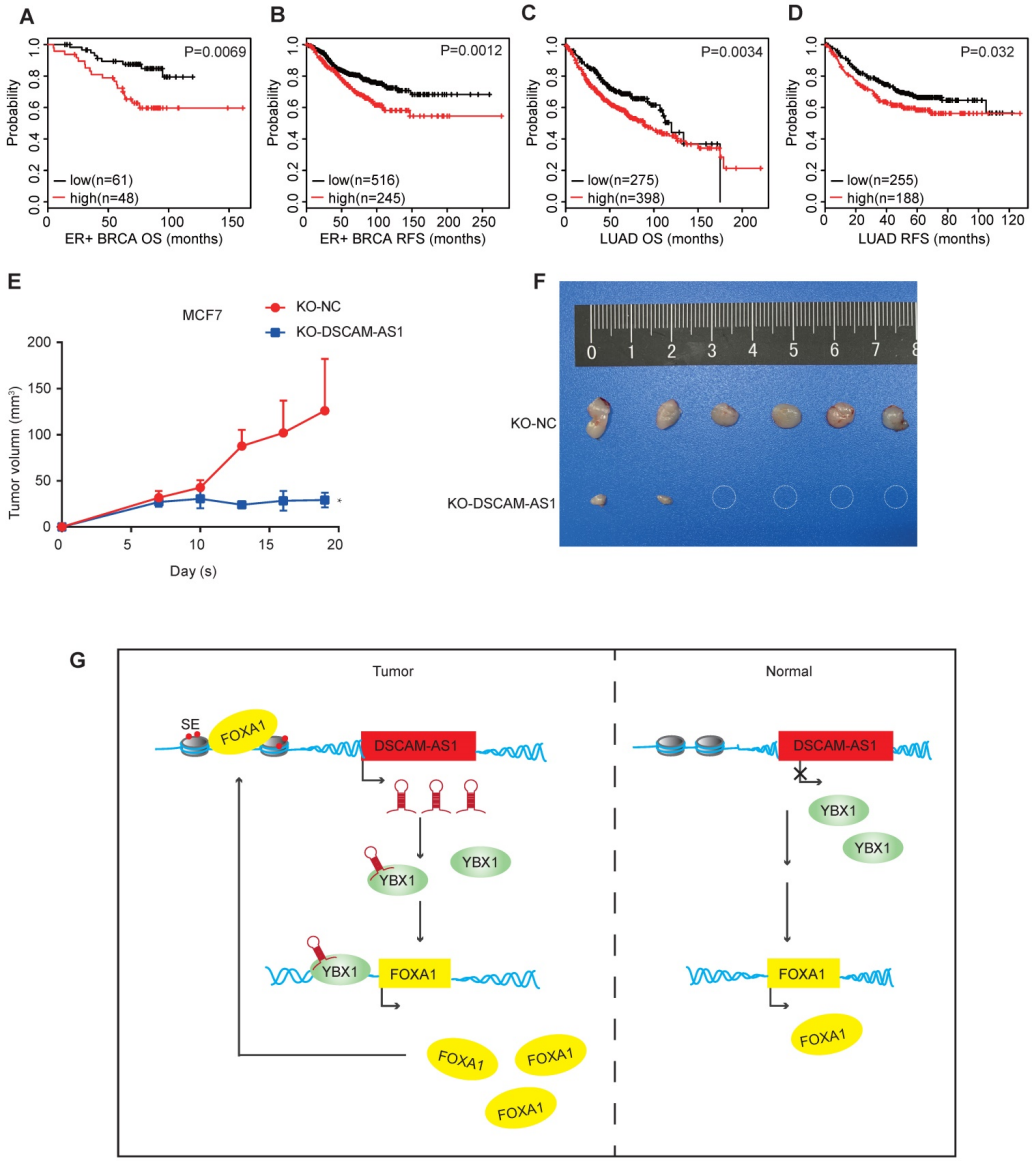
** DSCAM-AS1 is a potential biomarker and therapeutic target.** (**A, C**) Kaplan-Meier plots showed the association between DSCAM-AS1 expression levels and overall survival in ER+ breast cancer (A) and lung adenocarcinoma (C). (**B, D**) Kaplan-Meier plots indicating the association between DSCAM-AS1 expression levels and relapse-free survival in ER+ breast cancer (B) and lung adenocarcinoma (D). (**E**) Growth curve of nude mice inoculated with wild type (KO-NC) or KO-DSCAM-AS1 MCF7 cells. (**F**) Representative image of tumors at the end point. (**G**) Proposed working model showed DSCAM-AS1 could cooperate with YBX1 to promote the expression of FOXA1 and ERα.

Since the expression of DSCAM-AS1 was dramatically elevated in many breast cancer and lung adenocarcinoma patients, we then investigated the clinical relevance of the altered expressions of DSCAM-AS1. A probe which specifically targets DSCAM-AS1 in a U133 Plus 2.0 microarray platform allows us to survey the expression in many pieces of public data. To explore the clinical relevance of DSCAM-AS1 expression, we conducted Kaplan-Meier analysis in lung adenocarcinoma and breast cancer using the KM-plotter database 60. Higher expression of DSCAM-AS1 was significantly correlated with poor survival of ER^+^ breast cancer patients, where both the overall survival (OS) and relapse-free survival (RFS) were shorter than patients with low expression of DSCAM-AS1 (Figure 6A-B, *p<*0.05). Similar results were observed in lung adenocarcinoma (Figure 6C-D, *p<*0.05). Additionally, we analyzed the clinical relevance of DSCAM-AS1 interacting protein YBX1. High expression of YBX1 was found to correlated with bad prognosis (Figure S2I-L). To study the roles of DSCAM-AS1 *in vivo*, we conducted xenograft assays using MCF7 DSCAM-AS1 KO cells. We demonstrated DSCAM-AS1 depletion has profound effects on growth of MCF7 xenograft tumor (Figure 6E-F). Compared to negative control cells, the DSCAM-AS1 knockout cells showed a significant lower tumor formation rate (Figure 6E-F).

## Discussion

FOXA1 is an activated oncogenic transcription factor and acts as a crucial oncogene in the development of a variety of cancers like PRAD, BRCA, and LUAD by modulating a number of protein-coding genes that are involved in various cellular processes. FOXA1 is a pioneer factor that could increase accessibility of chromatin and enhance the recruitment of other transcription factors into its sites 61. It plays significant roles in gene regulation and morphogenesis and tumorigenesis in breast, prostate and lung cancers 2-4. We wondered if lncRNAs could also participate in the FOXA1 signaling pathway. Aberrant expression of lncRNAs has already been shown to be involved in biological processes and link to human diseases especially cancer. A large number of lncRNAs are still under-identified. We hypothesized that lncRNAs resulting from FOXA1 overexpression could also play a vital role in the development of the indicated cancers. Therefore, we aimed to extend the regulatory network of FOXA1 by investigating FOXA1 inducible lncRNAs. Using this principle, we identified 71 lncRNAs up-regulated by FOXA1 in PRAD, BRCA, and LUAD and more than 200 lncRNAs down-regulated in T47D breast cancer cell lines after the silencing of FOXA1.

In this study, we reported that DSCAM-AS1, as a FOXA1 regulated lncRNA, is able to physically interact with YBX1 and regulate expression of FOXA1 and ERα. DSCAM-AS1 shows a positive correlation with FOXA1 in TCGA samples and in CCLE cell lines. Furthermore, the promoter region of DSCAM-AS1 is remarkably occupied by FOXA1, suggesting that DSCAM-AS1 is potentially regulated by FOXA1. Therefore, DSCAM-AS1 is important in the regulation of FOXA1 functions. DSCAM-AS1 expression is elevated specifically in indicated types of human cancers, BRCA, LUAD, and PRAD, which shows that DSCAM-AS1 functions as an oncogenic molecule.

DSCAM-AS1 was first identified in ER^+^ breast cancer cells by Liu *et al.* 62 using suppression subtracted cDNA libraries in 2002. Niknafs* et al.* 22 and Minao *et al.* 23 discovered that DSCAM-AS1 was under transcriptional regulation by ERα, and over-expression of DSCAM-AS promotes proliferation, invasion and tamoxifen resistance in breast cancer 22. It has also been reported that DSCAM-AS1 was over-expressed and promoted cell invasion in NSCLC 24. However, the reason for its distinctive expression in breast cancer, lung adenocarcinoma and prostate cancer remains unknown. Therefore, in this study, we attempted to explore the reason and identify transcriptional factor that account for its distinctive expression. We revealed that FOXA1 transcriptionally activates DSCAM-AS1, which is important for its lineage-specific expression. First, DSCAM-AS1 was previously reported to be regulated by ERα 22, 23, 62, 63, and FOXA1 and ERα usually bind together to regulate their target genes in breast cancer, so the dominant expression of DSCAM-AS1 in ER^+^ breast cancer may be caused by cooperative regulation of FOXA1 and ERα. The second, FOXA1 was also reported to be capable to directly bind condensed chromatin and bind half of ERα binding sites independent of estrogen receptor 23, 61. This may be reason for the high expression of DSCAM-AS1 in some ER^-^ breast cancer cell lines, such as SKBR3 and MDA-MB-453 (Figure S1C), which have been reported as FOXA1 positive cells 9. Silencing of FOXA1 in SKBR3 cells significantly reduced the expression of DSCAM-AS1 (Figure 2D). Third, besides breast cancer, DSCAM-AS1 was also highly-expressed in lung adenocarcinoma and prostate cancer, in which ERα levels are low. Silencing ERα also resulted in no significant change of DSCAM-AS1 in lung adenocarcinoma (Figure S1I). Fourth, we observed two SEs near DSCAM-AS1, which is consistent with previous reporting 25. The pioneer factor FOXA1 was demonstrated to increase accessibility of chromatin and be vital for activation of the super-enhancers 64. Our analysis showed overlap between the FOXA1 and H3k27ac signal, indicating that DSCAM-AS1 is regulated by super-enhancers driven by FOXA1.

As for high abundance and lineage-specific expression of DSCAM-AS1, we also conducted analysis for its clinical relevance. Higher expression of DSCAM-AS1 was significantly correlated with poor survival of ER^+^ breast cancer patients. During the preparation of this manuscript, Elhasnaoui *et al.* 65 reported a re-analysis of DSCAM-AS1 effect on survival in BRCA, resulted in a conclusion similar to ours. Intriguingly, in lung cancer, DSCAM-AS1 showed distinctive expression in LUAD but was almost undetectable in LUSC (Figure S1B). Although FOXA1 exhibits significantly higher levels in LUAD than LUSC (Figure S1A), many LUSC samples have relatively high FOXA1 levels. These results indicate that there may be another TF which cooperatively transcriptionally activates DSCAM-AS1 with FOXA1. This TF might be distinctively expressed in lung adenocarcinoma. NKX2-1 (NKX homeobox-1 gene), also known as TTF-1 (thyroid transcription factor-1), is a TF that is specifically expressed in the lungs, thyroid, and ventral forebrain 66. NKX2-1 has been used as a molecular marker for lung adenocarcinoma in clinical work, especially for diagnosis of metastatic sites. NKX2-1 was also reported to cooperate with FOXA1 to regulate downstream genes 67. So, we tested whether NKX2-1 mediated the specific expression of DSCAM-AS1 in lung adenocarcinoma. However, after successfully silencing NKX2-1, we failed to observe a decrease of DSCAM-AS1 levels (Figure S5A-C). Further research is needed to explore the cooperative transcriptional factor mediates distinctively expressed of DSCAM-AS1 in lung adenocarcinoma.

Although the oncogenic role of DSCAM-AS1 in breast cancer has been reported 22-24, the molecular mechanisms remain to be further explored. In this study, we revealed two functional targets: FOXA1 and ERα. By interacting with YBX1, DSCAM-AS1 could enhance the recruitment of YBX1 in the promoter regions of FOXA1 and ERα. Our study revealed that DSCAM-AS1 forms a positive feedback loop with FOXA1 and ERα. Our study suggested that a FOXA1 and DSCAM-AS1 loop might be important for tumor growth of lung adenocarcinoma and breast cancer. ERα is the major mediator of the effects of estrogen and a crucial target of ER^+^ breast cancer, which accounts for 50-70% of breast cancers. Our studies showed DSCAM-AS1 can regulate expression of ERα. Silencing of DSCAM-AS1 significantly reduced the expression of ERα.

Pioneer factors like FOXA1 not only drive tumorigenesis but also participate in maintaining the malignant characteristic of tumors, thus making FOXA1 a potential therapeutic target for cancers 68. Unfortunately, though lots of efforts have been made towards the application of insights into pioneer factors, researchers have failed to identify inhibitors targeted against these pioneer factors in cancer, and they are still considered “un-druggable”. Consequently, the application of effectors involved in FOXA1 regulatory network seems essential. In this study, we revealed that DSCAM-AS1, as a direct FOXA1 target, plays an oncogenic role and is able to mediate the transcription of FOXA1. These results might provide another option that FOXA1 downstream genes like DSCAM-AS1 could be a potential therapeutic target, but much more follow-up studies are needed to support this hypothesis.

## Conclusion

In summary, our study identified 214 FOXA1 regulated lncRNAs, five of them showed significant over-expression in BRCA, PRAD and LUAD. We chose DSCAM-AS1 for detailed investigation. We demonstrated that DSCAM-AS1 was transcriptionally activated by super-enhancers driven by FOXA1 and exhibits lineage-specific expression patterns. We observed depletion of DSCAM-AS1 resulted in inhibiting cell growth and reducing FOXA1 and ERα expression. Mechanistically, DSCAM-AS1 was found to interact with YBX1 and enhance recruitment of YBX1 in the promoter regions of FOXA1 and ERα (Figure 6G). Moreover, DSCAM-AS1 may serve as a novel prognostic marker and potential therapeutic target.

## Supplementary Material

Supplementary figures and tables.

## Abbreviations

TSS: transcription start site
SE: super-enhancer
NSCLC: non-small cell lung cancer
LUAD: lung adenocarcinoma
LUSC: lung squamous cell carcinoma
BRCA: breast cancer
PRAD: prostate cancer
FOXA1: forkhead box A1
ERα: estrogen receptor α
ChIP: Chromatin immunoprecipitation
RIP: RNA Immunoprecipitation
MTT: 3-(4,5-dimethylthiazol-2-yl)-2,5-diphenyltetrazolium bromide
OS: Overall survival
RFS: relapse-free survival
KO: knock out
siRNAs: Small interfering RNAs
TCGA: The Cancer Genome Atlas
CCLE: Cancer Cell Line Encyclopedia
ENCODE: Encyclopedia of DNA Elements
YBX1: Y box binding protein 1
TF: transcription factor
CNVs: copy number variations
EMT: epithelial to mesenchymal transition
