# Supplementary material for: LncRNA DSCAM-AS1 interacts with YBX1 to promote cancer progression by forming a positive feedback loop that activates FOXA1 transcription network
Source: Theranostics. 2020 Aug 29;10(23):10823–37. doi: 10.7150/thno.47830 (PMC7482804; doi:10.7150/thno.47830)
Supplement: Supplementary file 1 — Supplementary figures and tables. [file thnov10p10823s1.pdf]

Fig S1 The correlation of FOXA1 and DSCAM-AS1 expression in BRCA and LUAD

(A) The expression pattern of FOXA1 in lung cancer patients.

(B) The detailed expression level of DSCAM-AS1 in normal and tumors of LUAD and LUSC.

(C) DSCAM-AS1 expression levels of different cancer cell lines from CCLE database.

(D) qPCR showed the relative expression of DSCAM-AS1 in different cancer cell lines.

(E) Pan cancer analysis of CNV status of DSCAM-AS1.

The CNVs' status were analyzed using cBioPortal database (http://www.cbioportal.org/) and COSMIC dataset (V74). The cBioPortal database which is developed by Memorial Sloan Kettering Cancer Center (MSK). It is a comprehensive and widely-used platform that allows data mining, data integration based on the TCGA database. We analyzed the correlation between copy number variation and gene expression.

As shown in the result, only very small proportion of samples (< ~1%) occurs DSCAM-AS1 amplification in BRCA and LUAD. But the proportion of samples with DSCAM-AS1 overexpression (as mentioned above, we considered the FPKM >2 as high expression) is 33% (276/844) and 19% (93/488) for BRCA, and LUAD, respectively. Since there is no detailed sample ID information in result from cBioPortal database, we further analyzed by using COSMIC dataset (BRCA n=844; LUAD n=488). For breast cancer, 0.7% (6/844) were found to have DSCAM-AS1 amplification, but only 3 cases of amplification were found in high DSCAM-AS1 expression samples (n=276). For lung adenocarcinoma, only 1/488 case of amplification was found in high DSCAM-AS1 expression samples (n=93). These data indicated that the over-expression of DSCAM-AS1 is not associated with gene amplification in BRCA and LUAD (p >0.05, Fisher exact test).

(F) The expression of host gene DSCAM was detected in MCF7 cells treated by different concentration of JQ1.

(G) Potential transcriptional factors that bind at the promoter region of DSCAM-AS1 from ChIPBase database.

(H) Motif analysis was conducted by JASPR showing potential binding sites at the promoter region of different transcription factors.

(I) Knockdown of ERα in lung cancer cell line NCI-H1437, the expression of DSCAM-AS1 was detected by RT-qPCR.

The expression levels of FOXA1 and DSCAM-AS1 in MCF7 (J), T47D (L), SKBR3 (M), NCI-H1437 (N), NCI-H1573 (P), 22Rv1 (Q) cell lines after transfecting FOXA1 siRNAs.

(K) Western Blot showing the protein level of FOXA1 after silencing of FOXA1 in MCF7 cell.

(O) Western Blot showing the protein level of FOXA1 after silencing of FOXA1 in NCI-H1437 cell.

Fig S2 DSCAM-AS1 regulated cell cycle and regulated the expression of ERα

(A) Flow cytometry was performed after silencing DSCAM-AS1 in MCF7.

(B) Cell cycle was detected by flow cytometry in DSCAM-AS1 knockdown in T47D.

(C) RT-qPCR assays showed the knockdown efficiency of DSCAM-AS1 by siRNAs in MCF7.

(D) RT-qPCR assays showed the knockdown efficiency of DSCAM-AS1 by siRNAs in T47D.

(E) Knock out of DSCAM-AS1 by CRISPR/Cas9 in MCF7 and NCI-H1437, the expression of DSCAM-AS1 was detected by RT-qPCR.

(F) The expression of host gene DSCAM was detected after knock-out of DSCAM-AS1 using CRISPR/Cas9 method.

The expression levels of ERα were detected by western blot (G) and RT-qPCR (H) methods after knockout of DSCAM-AS1 using CRISPR/Cas9

Kaplan-Meier plots indicating the association between YBX1 expression level and relapse-free survival in ER^+ breast cancer (I&J) and lung adenocarcinoma (K&L).

Fig S3 SURF6 did not affect the expression of DSCAM-AS1 and had no effect on cell growth

RT-qPCR showed the expression levels of DSCAM-AS1 after silencing of SURF6 using two individual siRNAs in MCF7 (A) and NCI-H1437 (B) cells.

MTT assay was used to detect the effect on cell proliferation after silencing of SURF6 in MCF7 (C) and NCI-H1437 (D) cells.

Fig S4 DSCAM-AS1 interacts with YBX1

(A) RT-qPCR assay showed the enrichment of different DSCAM-AS1 isoforms.

(B) The annotation of DSCAM-AS1 isoforms.

(C) ChIP-seq data showed the enrichment of YBX1 in the promoter region of FOXA1 and ERα.

Fig S5 The correlation of NKX2-1 and DSCSAM-AS1 in lung adenocarcinoma

(A) Knockdown of NKX2-1 in lung cancer cell line NCI-H1437, the expression of DSCAM-AS1 was detected by RT-qPCR.

(B) Knockdown of NKX2-1 in lung cancer cell line NCI-H1573, the expression of DSCAM-AS1 was detected by RT-qPCR.

(C) Knockdown of NKX2-1 in lung cancer cell line NCI-H2110, the expression of DSCAM-AS1 was detected by RT-qPCR.

Supplementary Figure 1

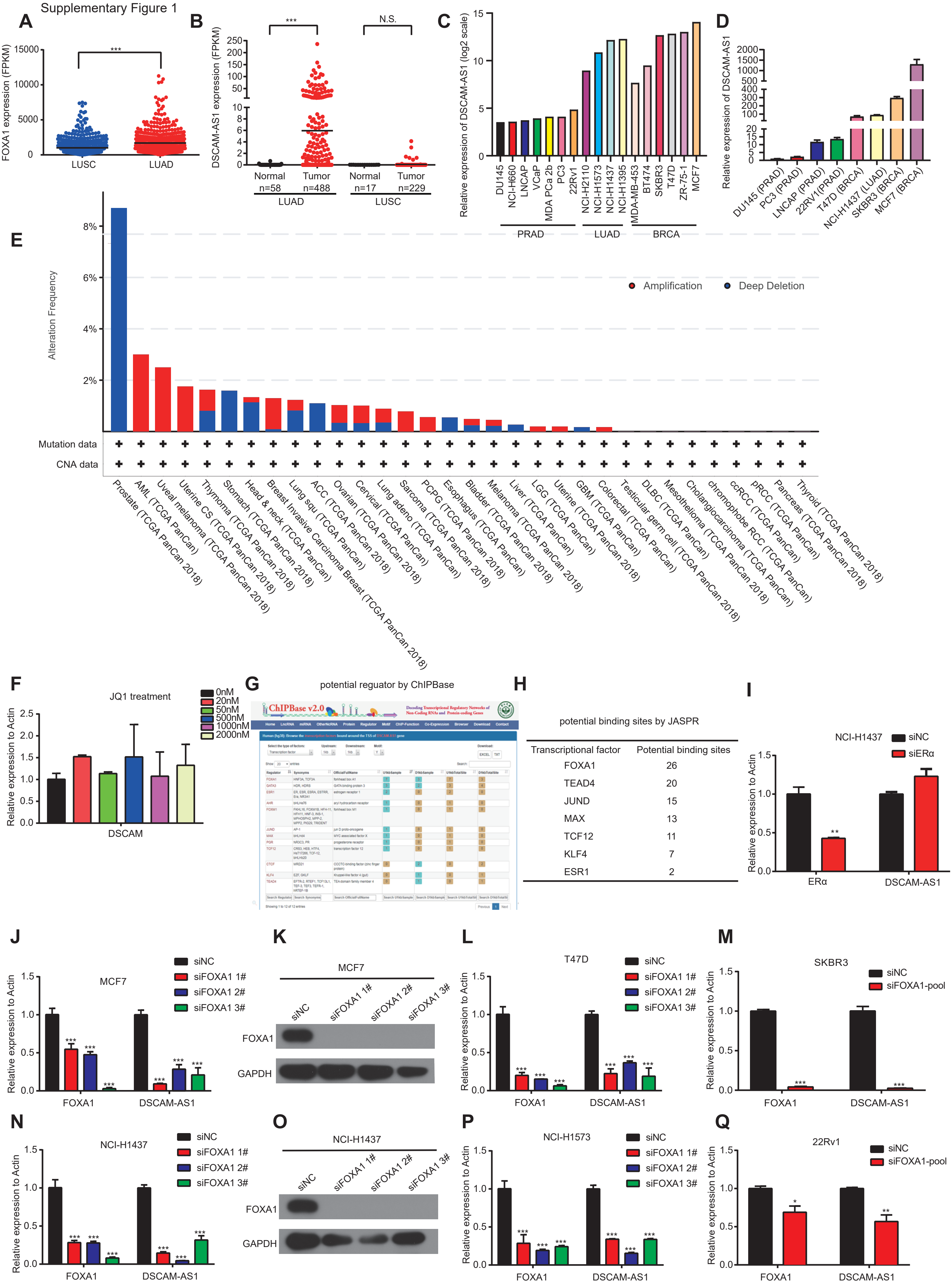

Supplementary Figure 2

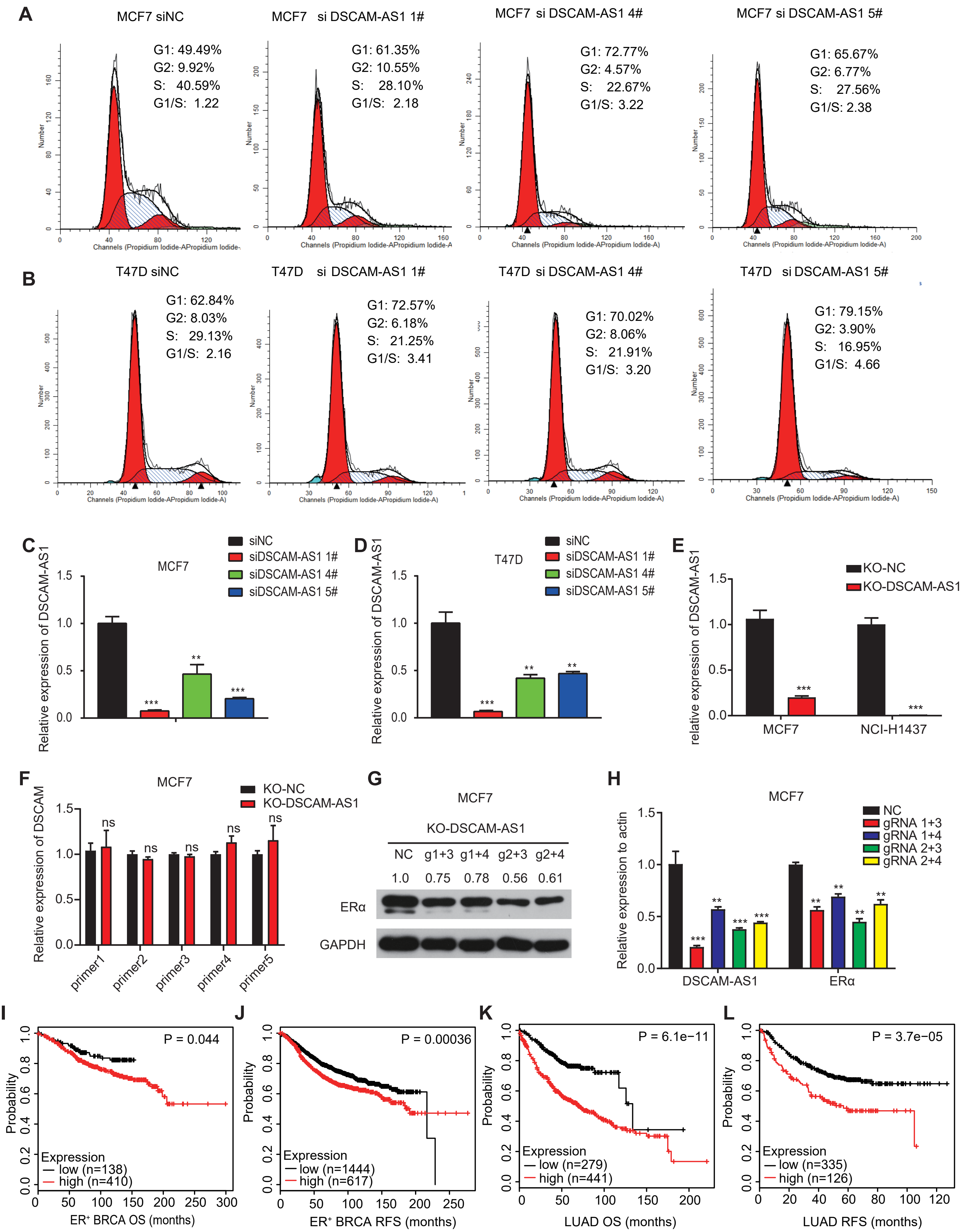

Supplementary Figure 3

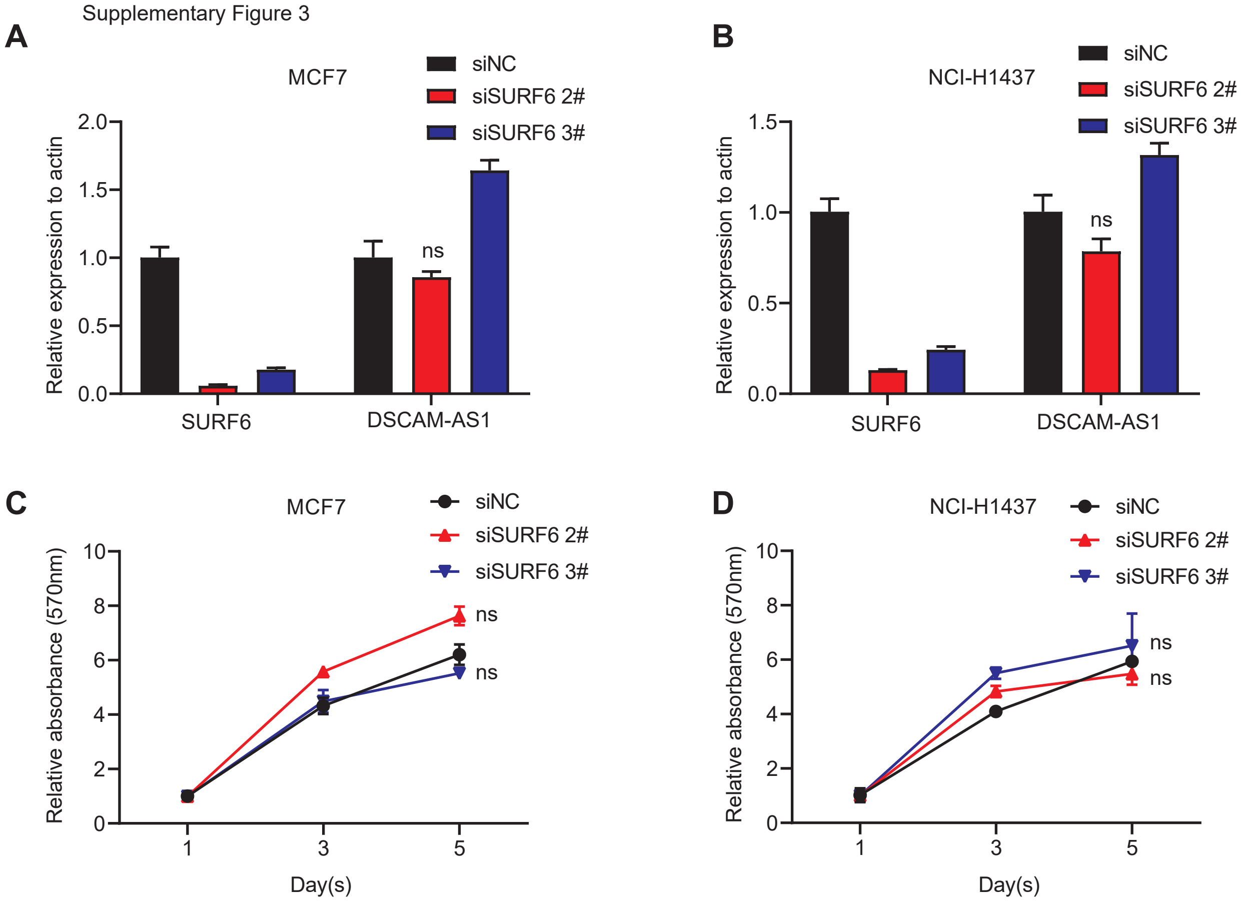

Supplementary Figure 4

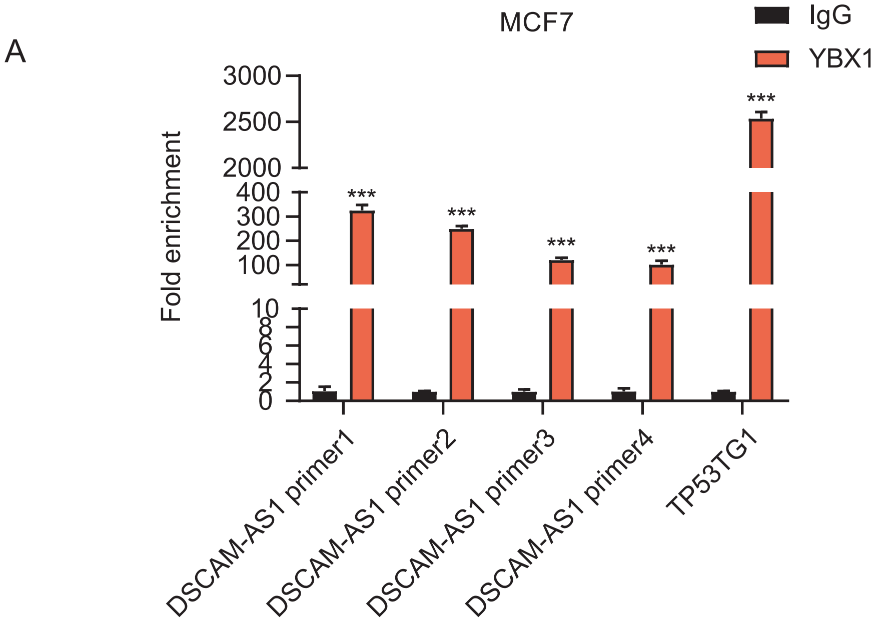

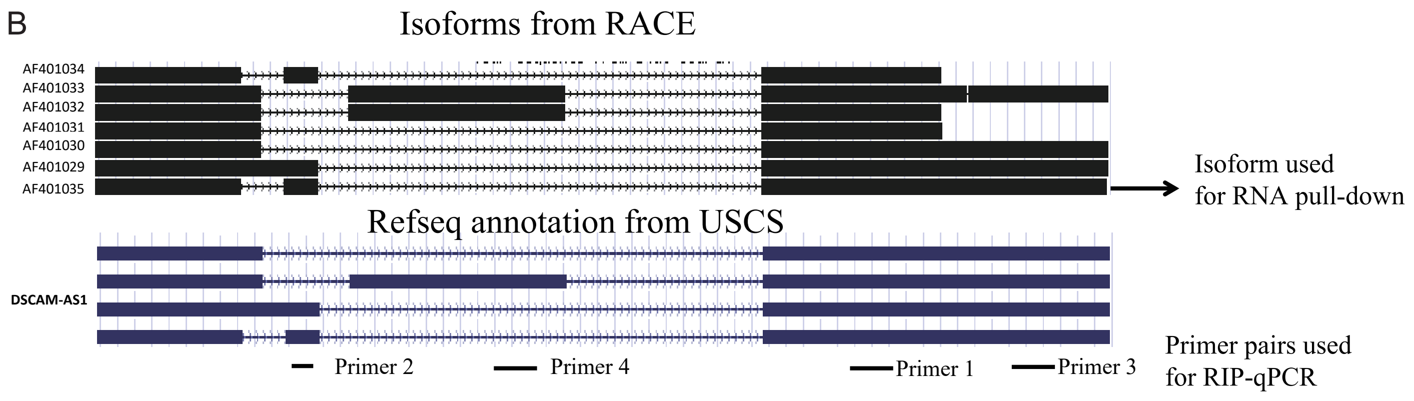

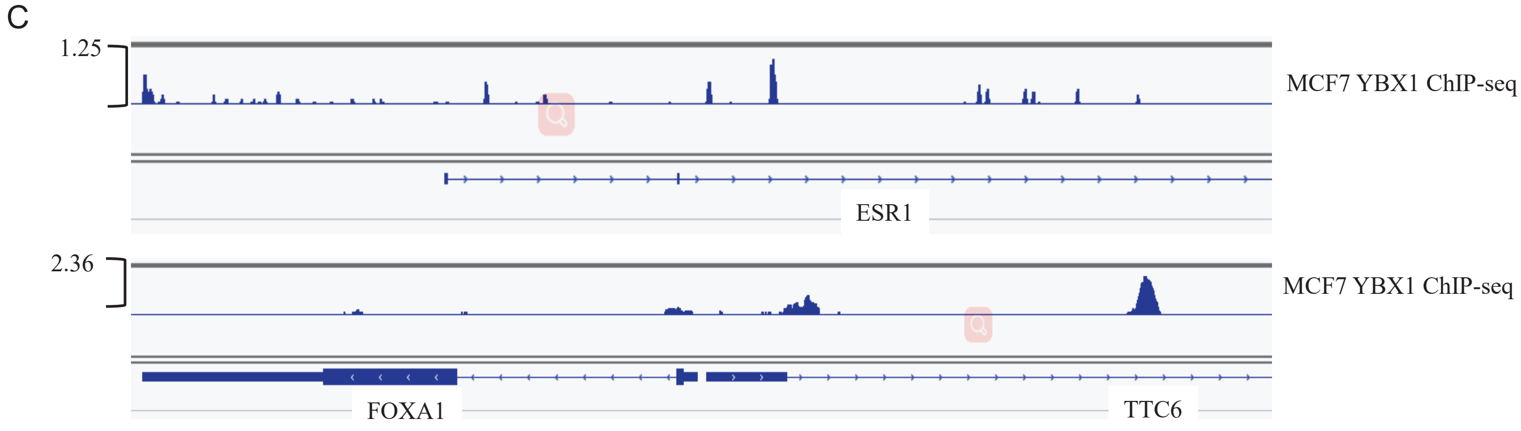

Supplementary Figure 5

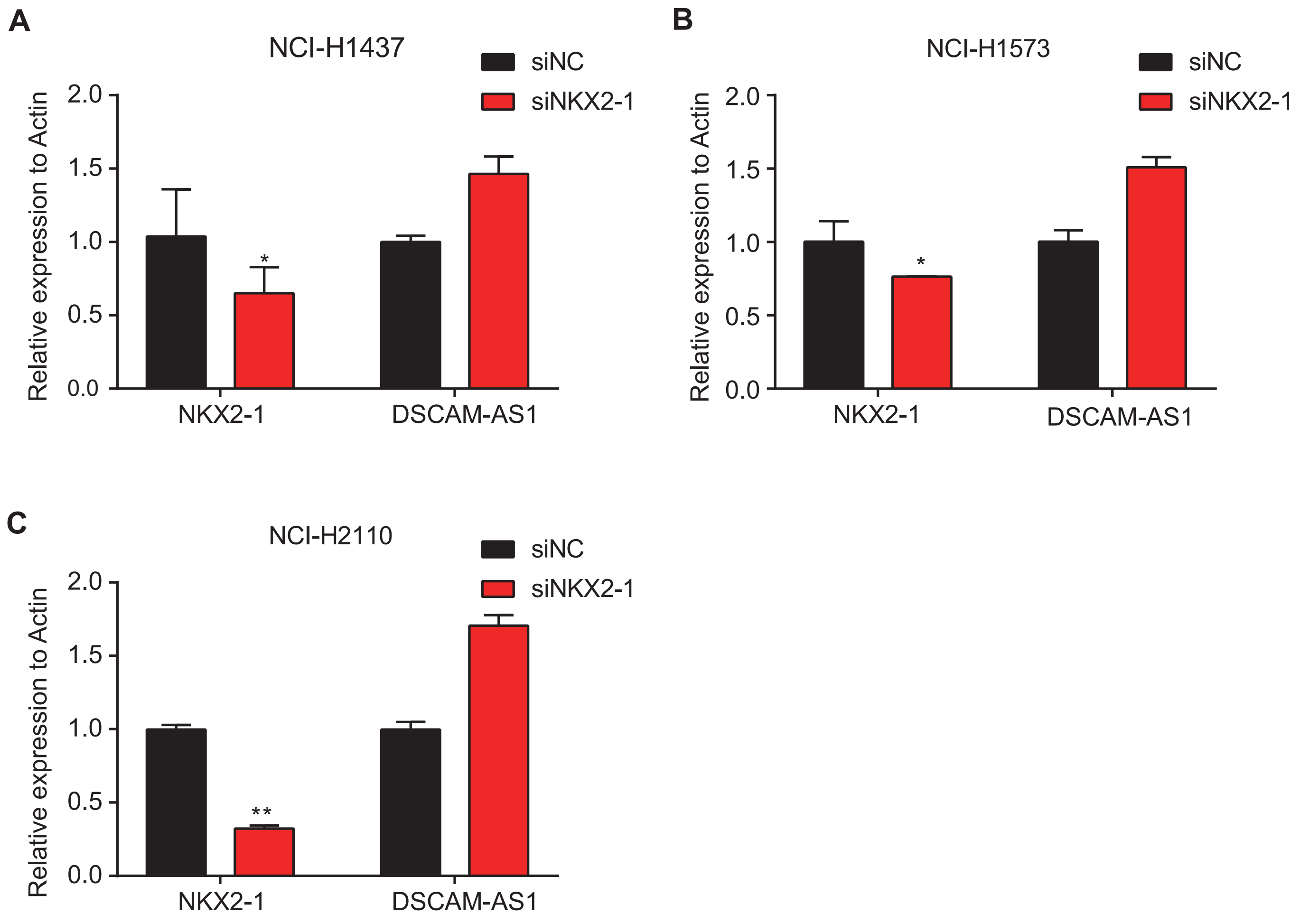

Table S1. Primers used in this study.

Genes	Forward primer (5‘-3’)	Reverse primer (5‘-3’)
RT-qPCR		
DSCAM-AS1	ACAGAGATGGACGACGGATC	TTGTGAGCCTGAGAGATCCC
Actin	TCGTGCGTGACATTAAGGAG	GTCAGGCAGCTCGTAGCTCT
DSCAM	CCTACGAACACGCCAAGATG	TACTCATTTGTCCCTGCCGT
C-MYC	TTTCGGGTAGTGGAAAACCA	CACCGAGTCGTAGTCGAGGT
NKX2-1	CCAAACTTCACTCTGGGCAC	CTCTTTCCTCTCCCCAGCTC
FOXA1	ACAGCTACTACGCAGACACG	CCCAGGCCTGAGTTCATGTT
YBX1	CGGAGGCAGCAAATGTTACA	GACCCCTACGACGTGGATAG
ERα	ATGATCAACTGGGCGAAGAG	CAGGATCTCTAGCCAGGCAC
MEG3	GGCCTCTCGTCTCCTTCCT	GGGTCCCACATTCGAGGTC
DSCAM-primer1	ATCAGACCCAGCGAACTCAG	CCAGCGGTAATCTGGCTCAG
DSCAM-primer2	CCTACGAACACGCCAAGATG	TACTCATTTGTCCCTGCCGT
DSCAM-primer3	ACGTTAAGCAAGCAACAGCA	TCTCCAAAGTCAGCATCCGT
DSCAM-primer4	TCCGCCTTTCATACAACCCT	TTCTGCCAGGTGATCGTGAT
DSCAM-primer5	ACCTGAACATCTCCAGCTCC	TGCAGGCCCTCTTACGTTTA
SURF6	TCCGGAAGCGAGAAGAGAAG	CCCAAGCTGCTTCCTCTTTG
DSCAM-AS1-primer1	ACAGAGATGGACGACGGATC	TTGTGAGCCTGAGAGATCCC
DSCAM-AS1-primer2	AGCCACAGAAGGTACCAGTT	GCTGAGAATTCTAGTGGAGGC
DSCAM-AS1-primer3	CACACACTCCCACACATAGC	ACGTTGGATGGGAAAAGTGA
DSCAM-AS1-primer4	ACTGTGTTCACGATGCTTCC	ACTGCGTGTTTCCTAGTCCC
TP53TG1	CTTTCCTTTAATCTTCGGAGGC	TGCCAGCTCTCAGAGTCCTT
ChIP-qPCR		
DSCAM-AS1-P1	GAAGGAAAGGAAACATACCATAAA	TGTCGAATTCCTGGCCTTAG
DSCAM-AS1-P2	AGTGCAAACAATTGCAAAACA	GACCTGCCCAGCCAGATA
DSCAM-AS1-P3	GACATGGCAGGGACAAGAGT	CCGATCTCAGGGAAACAAAC
DSCAM-AS1-P4	GAGTGGAACCCACTGATGGT	CTGCAAAAACGTGCTGAGAA

DSCAM-AS1-P5	TCACAGCCAAGGAAACACAG	TCACCTCTGCCTCCCTTATG
FOXA1-P1	AACAAGAAGATTTTGCGGATTC	GACCGGGTGATAGGCAATTA
FOXA1-P2	AACTGCACATCTTCACCCTC	TGTGTTTTAACTTGTCTTCC
ERα-P1	GCACACCCCATTCTATCTGC	CCGGCTTTCTCTAATGTGCT
ERα-P2	CAGCAGCGACGACAAGTAAA	TACTGGTCTCCCGAGCTCAT
PCR		
DSCAM-AS1 for tRSA pull down	CCGGATATCCTTTGGGAGGCTGAGGCAGG	AAATATGCGGCCGCTAAATGCATGCTTGATGGAG

Table S2. sgRNAs used in CRIPSR/Cas9

**sgRNAs**	**sequences**
sgRNA1	ACTAGGGGACTTGCTAAAGC
sgRNA2	ACGAGGGACCGCATCTGACA
sgRNA3	CAACAAATCACTAGTACCGC
sgRNA4	CGTCTCTTATGTGGGTGCCG

Table S3. siRNAs used in this study

siRNAs	Sense (5‘-3’)	Antisense (5‘-3’)
siDSCAM-AS1 1#	GUUCUGGUCUCAUCAUGAUTT	AUCAUGAUGAGACCAGAACTT
siDSCAM-AS1 4#	CCUCCUCCAACUGCCAUUUTT	AAAUGGCAGUUGGAGGAGGTT
siDSCAM-AS1 5#	CACAUAGGCAUGACAUACUTT	AGUAUGUCAUGCCUAUGUGTT
siFOXA1 1#	GAGAGAAAAAAUCAACAGCTT	GCUGUUGAUUUUUUCUCUCTT
siFOXA1 2#	CACACAAACCAAACCGUCATT	UGACGGUUUGGUUUGUGUGTT
siFOXA1 3#	CCGGUCAGCAACAUGAACUTT	AGUUCAUGUUGCUGACCGGTT
siYBX1 1#	GGAUAUGGUUUCAUCAACATT	UGUUGAUGAAACCAUAUCCTT
siYBX1 2#	CGUAACCAUUAUAGACGCUTT	AGCGUCUAUAAUGGUUACGTT
siYBX1 3#	CCGGUUUAGUCAUCCAACATT	UGUUGGAUGACUAAACCGGTT
siERα 1#	GAAUGUGCCUGGCUAGAGATT	UCUCUAGCCAGGCACAUUCTT
siERα 2#	CGAGUAUGAUCCUACCAGATT	UCUGGUAGGAUCAUACUCGTT
siNKX2-1 1#	CGCCGUACCAGGACACCAUTT	AUGGUGUCCUGGUACGGCGTT
siNKX2-1 3#	GCACACGACUCCGUUCUCATT	UGAGAACGGAGUCGUGUGCTT
siNKX2-1 3#	UGAAGCGCCAGGCCAAGGATT	UCCUUGGCCUGGCGCUUCATT

Table S4 Public dataset used in this study

Dataset type	Accession	Reason for choosing dataset
FOXA1 ChIP-seq	GSM631469; GSM631470; GSM631473; GSM1858657	High quality FOXA1 ChIP-seq data of MCF7, T47D cell lines (with biological duplicates).
ERα ChIP-seq	GSM1669042; GSM1669011	High quality ERα ChIP-seq data of MCF7, T47D cell lines
H3K27ac ChIP-seq	GSM1382473; GSM1382472; GSM1382482; GSM1382483; GSM1693016; GSM1589470; GSM2258849; GSM1589474; DRR017019; DRR017127; DRR016965	High quality H3K27ac ChIP-seq data of MCF7, T47D (ER^+ BRCA), MDA-MB-468, MDA-MB-231 (ER^- BRCA), NCI-H2126, RERF-LC-KJ, NCI-H1437 (LUAD with high expression of DSCAM-AS1) cell lines.
si-FOXA1 RNA-seq	GSM2231402; GSM2231403; GSM2231408; GSM2231409	High quality of RNA-seq data of T47D cells treated with control siRNA and FOXA1 siRNA (with biological duplicates).
Hi-C data	GSM2827515; GSM2827516	ENCODE Project Hi-C data of T47D (with biological duplicates).

Table S5 FOXA1 regulated lncRNAs

lncRNA ID	lncRNA name	log2 Fold change	FDR
ENSG00000248740	RP11-328K4.1	-8.03	4.34E-17
ENSG00000235263	RP13-392I16.1	-7.17	1.15E-09
ENSG00000229352	AC007563.3	-6.89	8.44E-08
ENSG00000225667	LINC00505	-6.77	3.27E-07
ENSG00000234693	RP4-530I15.6	-6.77	3.28E-07
ENSG00000254967	RP11-680F20.6	-6.59	3.52E-06
ENSG00000249392	RP11-725D20.1	-6.19	1.31E-39
ENSG00000248771	LINC01207	-6.14	0.00010757
ENSG00000236039	AC019117.2	-6.05	0.00021086
ENSG00000243885	RP11-278L15.2	-6.04	0.00021267
ENSG00000234111	RP11-364P22.1	-5.84	0.00078007
ENSG00000203721	LINC00862	-5.77	6.05E-17
ENSG00000241720	RP4-735C1.4	-5.69	2.02E-89
ENSG00000244468	RP11-206M11.7	-5.09	6.26E-259
ENSG00000258752	RP11-356K23.1	-5.06	3.29E-09
ENSG00000269246	CTC-246B18.10	-5.06	2.74E-09
ENSG00000267924	RP11-255H23.4	-4.93	1.66E-08
ENSG00000259420	RP11-307C19.2	-4.91	1.49E-15
ENSG00000260228	RP11-483P21.2	-4.88	2.71E-207
ENSG00000257060	RP11-26608.1	-4.54	3.12E-16
ENSG00000250508	RP11-757G1.6	-4.44	1.46E-118
ENSG00000272865	RP11-561I11.4	-4.35	2.60E-05
ENSG00000269900	RMRP	-4.33	1.01E-33
ENSG00000263586	HID1-AS1	-4.26	1.46E-09
ENSG00000238117	AP004372.1	-4.26	1.74E-231
ENSG00000256357	RP11-349I1.2	-4.18	6.32E-09
ENSG00000244541	LINC01213	-4.14	0.00015256
ENSG00000269420	PLA2G4C-AS1	-4.14	9.09E-09
ENSG00000259450	RP11-265N7.1	-4.14	8.11E-08
ENSG00000251526	RP11-381N20.1	-4.13	0.00045505
ENSG00000253298	AC008703.1	-3.99	0.0006659
ENSG00000259669	RP11-643A5.2	-3.98	0.00051397
ENSG00000226644	RP11-128M1.1	-3.90	0.00094276
ENSG00000258414	RP11-35609.1	-3.88	4.60E-87
ENSG00000259001	RPPH1	-3.87	1.21E-11
ENSG00000230943	RP11-367G18.1	-3.70	3.17E-05
ENSG00000227075	AP000472.3	-3.69	4.81E-34
ENSG00000250891	CTD-2281M20.1	-3.63	7.10E-13
ENSG00000267107	PCAT19	-3.57	1.63E-07
ENSG00000244252	RP11-495P10.7	-3.52	0.00016901
ENSG00000257002	AP000438.2	-3.50	1.30E-33
ENSG00000261754	CTC-523E23.1	-3.39	2.38E-16
ENSG00000262179	RP1-302G2.5	-3.38	0.00045603
ENSG00000243583	RP4-669B10.3	-3.36	6.99E-24
ENSG00000235491	AC097499.1	-3.35	9.83E-08
ENSG00000266402	SNHG25	-3.35	1.64E-80
ENSG00000248663	LINC00992	-3.33	2.68E-234
ENSG00000260912	RP11-363E7.4	-3.32	2.03E-89
ENSG00000260997	RP4-647J21.1	-3.31	1.53E-07
ENSG00000271850	RP11-16D22.2	-3.31	1.57E-07
ENSG00000269289	CTB-92J24.3	-3.30	2.12E-17

ENSG00000255727	LINC01489	-3.16	5.99E-17
ENSG00000214708	AC090616.2	-3.15	8.48E-132
ENSG00000272941	RP11-134L10.1	-3.13	2.39E-11
ENSG00000225652	KCNMA1-AS3	-3.11	4.72E-05
ENSG00000245750	DRAIC	-3.01	4.40E-30
ENSG00000265246	RP11-663N22.1	-2.99	1.79E-82
ENSG00000261538	RP11-3M1.1	-2.99	2.69E-20
ENSG00000272273	XXbac-BPG252P9.10	-2.92	2.74E-06
ENSG00000232023	AC009410.1	-2.90	5.82E-25
ENSG00000235123	DSCAM-AS1	-2.88	4.32E-153
ENSG00000267767	CTC-523E23.4	-2.87	8.78E-10
ENSG00000255427	RP11-350D17.2	-2.78	1.07E-09
ENSG00000185847	LINC01405	-2.74	1.81E-17
ENSG00000237267	LINC01519	-2.74	7.05E-23
ENSG00000256940	RP11-783K16.5	-2.73	1.07E-39
ENSG00000228741	SPATA13	-2.73	3.91E-14
ENSG00000272746	RP11-53B2.6	-2.61	1.43E-15
ENSG00000232767	RP11-498B4.5	-2.60	2.37E-07
ENSG00000240567	RP11-3P17.4	-2.58	6.55E-06
ENSG00000257193	RP11-818F20.4	-2.57	1.02E-23
ENSG00000259883	EHD4-AS1	-2.52	3.25E-10
ENSG00000269235	ZNF350-AS1	-2.49	3.85E-25
ENSG00000230978	LINC00160	-2.44	1.62E-05
ENSG00000273464	RP11-313P22.1	-2.42	2.92E-08
ENSG00000248429	RP11-597D13.9	-2.40	3.97E-106
ENSG00000261229	MTHFS	-2.40	1.04E-08
ENSG00000271109	CTC-523E23.11	-2.38	3.17E-28
ENSG00000231119	RP4-569M23.2	-2.38	8.50E-06
ENSG00000259485	CTD-2147F2.1	-2.37	4.70E-21
ENSG00000257553	RP11-603J24.17	-2.37	3.09E-05
ENSG00000255471	RP11-736K20.5	-2.37	2.46E-30
ENSG00000259702	RP11-236L14.1	-2.33	6.20E-17
ENSG00000266176	RP11-855A2.5	-2.33	3.26E-12
ENSG00000255794	RMST	-2.32	7.46E-12
ENSG00000257443	RP11-150C16.1	-2.31	3.00E-11
ENSG00000226965	AC003088.1	-2.30	6.23E-06
ENSG00000260136	CTD-2270L9.4	-2.27	8.59E-44
ENSG00000270571	RP11-355F16.1	-2.27	3.66E-05
ENSG00000271869	RP11-51J9.5	-2.24	9.84E-10
ENSG00000231908	IDH1-AS1	-2.21	8.75E-11
ENSG00000219545	UMAD1	-2.17	1.52E-43
ENSG00000249684	RP11-423H2.3	-2.15	2.72E-64
ENSG00000240032	RP11-274H2.3	-2.13	0.00018586
ENSG00000237457	LINC01351	-2.08	1.68E-07
ENSG00000230623	RP11-469A15.2	-2.08	2.16E-13
ENSG00000261051	RP11-274H2.5	-2.07	0.00034794
ENSG00000235978	AC018816.3	-2.07	2.72E-70
ENSG00000235501	RP4-639F20.1	-2.06	5.15E-25
ENSG00000254337	RP11-865I6.2	-2.05	1.01E-47
ENSG00000250303	RP11-356J5.12	-2.04	0.00022372
ENSG00000224189	HAGLR	-2.03	5.30E-12
ENSG00000273143	RP11-525A16.4	-2.02	0.00014112
ENSG00000152931	PART1	-2.01	4.86E-11
ENSG00000223478	RP11-545E17.3	-1.99	3.19E-41

ENSG00000260081	RP11-66N11.8	-1.96	2.34E-10
ENSG00000249395	CASC9	-1.96	2.34E-11
ENSG00000235939	RP11-123B3.2	-1.92	1.12E-35
ENSG00000272369	RP11-446N19.1	-1.91	5.53E-18
ENSG00000258053	CTD-2021H9.3	-1.87	7.16E-26
ENSG00000228661	AC090587.5	-1.86	0.0009609
ENSG00000235448	LURAP1L-AS1	-1.86	1.02E-16
ENSG00000255198	SNHG9	-1.86	4.47E-33
ENSG00000261645	DISC1FP1	-1.86	6.62E-05
ENSG00000229666	MAST4-AS1	-1.85	1.22E-07
ENSG00000249993	BFSP2-AS1	-1.85	3.95E-05
ENSG00000238005	RP11-443B7.1	-1.85	1.39E-10
ENSG00000259038	CTD-2325P2.4	-1.85	1.69E-13
ENSG00000273149	RP11-290D2.6	-1.84	5.62E-05
ENSG00000231482	AC141930.2	-1.81	1.71E-08
ENSG00000222041	LINC00152	-1.81	4.24E-52
ENSG00000267475	CTD-2538C1.2	-1.81	9.69E-30
ENSG00000233098	CCDC144NL-AS1	-1.79	0.00032835
ENSG00000237978	KCNMB2-AS1	-1.78	2.02E-42
ENSG00000223813	AC007255.8	-1.78	2.92E-23
ENSG00000236753	MKLN1-AS	-1.76	4.86E-24
ENSG00000257303	RP11-977G19.11	-1.76	3.50E-09
ENSG00000231806	PCAT7	-1.75	8.25E-12
ENSG00000177337	DLGAP1-AS1	-1.73	2.73E-19
ENSG00000257252	RP11-486A14.2	-1.68	0.00068968
ENSG00000247595	SPTY2D1-AS1	-1.67	6.07E-12
ENSG00000235079	ZRANB2-AS1	-1.66	1.80E-05
ENSG00000226471	CTA-292E10.6	-1.66	4.62E-18
ENSG00000261183	RP11-532F12.5	-1.65	1.98E-60
ENSG00000266921	RP11-15A1.7	-1.65	4.18E-05
ENSG00000186019	AC084219.4	-1.65	2.02E-11
ENSG00000267174	CTC-510F12.4	-1.65	8.65E-06
ENSG00000249916	CTD-2280E9.1	-1.64	2.53E-05
ENSG00000255458	RP11-539G18.3	-1.64	4.91E-05
ENSG00000260196	RP1-239B22.5	-1.64	2.11E-24
ENSG00000270673	YTHDF3-AS1	-1.63	6.82E-18
ENSG00000245648	RP11-277P12.20	-1.61	1.63E-37
ENSG00000268129	RP11-91G21.1	-1.60	1.87E-11
ENSG00000182648	LINC01006	-1.59	8.84E-11
ENSG00000269293	ZSCAN16-AS1	-1.59	4.28E-19
ENSG00000267284	RP11-397A16.1	-1.58	1.39E-07
ENSG00000273199	AP000692.10	-1.58	0.00016214
ENSG00000255507	RP11-535A19.2	-1.58	0.00059836
ENSG00000235453	TOPORS-AS1	-1.56	1.07E-15
ENSG00000245213	RP11-10K16.1	-1.54	2.44E-16
ENSG00000248664	CTC-498J12.3	-1.53	0.00010668
ENSG00000257261	RP11-96H19.1	-1.53	1.70E-33
ENSG00000232671	RP11-126K1.2	-1.52	2.28E-08
ENSG00000203706	SERTAD4-AS1	-1.51	7.38E-11
ENSG00000262117	BCAR4	-1.51	1.14E-08
ENSG00000259345	RP11-624L4.1	-1.50	5.38E-05
ENSG00000257027	RP11-705C15.3	-1.50	1.15E-14
ENSG00000237807	RP11-400K9.4	-1.49	2.73E-53
ENSG00000177410	ZFAS1	-1.48	5.63E-53

ENSG00000230490	RP11-141M1.3	-1.48	2.88E-06
ENSG00000266970	RP11-806H10.4	-1.46	1.58E-05
ENSG00000267702	RP11-53B2.2	-1.46	3.88E-11
ENSG00000232065	LINC01063	-1.43	0.00042543
ENSG00000247011	RP11-700H6.1	-1.43	0.00011302
ENSG00000250742	RP11-834C11.4	-1.42	4.09E-18
ENSG00000269933	RP3-333A15.2	-1.42	1.34E-08
ENSG00000236423	LINC01134	-1.42	0.00082806
ENSG00000254286	RP11-89K10.1	-1.40	1.06E-12
ENSG00000261804	RP11-44F14.2	-1.40	1.14E-16
ENSG00000258884	CTD-3035D6.2	-1.39	9.27E-10
ENSG00000270820	RP11-355B11.2	-1.36	2.04E-12
ENSG00000250522	AC004066.3	-1.35	0.00053817
ENSG00000249116	CTD-2194D22.3	-1.34	4.13E-05
ENSG00000229109	RP11-439K3.1	-1.34	2.31E-05
ENSG00000172965	MIR4435-2HG	-1.34	2.25E-20
ENSG00000225868	AC016582.2	-1.34	9.07E-05
ENSG00000214194	LINC00998	-1.34	1.32E-27
ENSG00000251169	AC005355.2	-1.33	0.00032673
ENSG00000273237	CTB-119C2.1	-1.33	1.42E-20
ENSG00000234327	AC012146.7	-1.31	1.96E-12
ENSG00000266602	RP11-476K15.1	-1.31	2.60E-24
ENSG00000272009	RP1-313I6.12	-1.30	1.79E-06
ENSG00000258704	SRP54-AS1	-1.27	4.67E-05
ENSG00000261189	RP3-512B11.3	-1.25	1.35E-08
ENSG00000254854	CTD-2523D13.2	-1.25	5.26E-10
ENSG00000260936	FTO-IT1	-1.23	5.17E-06
ENSG00000260708	CTA-29F11.1	-1.20	8.99E-20
ENSG00000260260	SNHG19	-1.20	9.55E-21
ENSG00000243350	RP11-379F12.3	-1.19	1.68E-05
ENSG00000224746	AC015987.1	-1.19	5.28E-06
ENSG00000223797	ENTPD3-AS1	-1.18	7.82E-20
ENSG00000269893	SNHG8	-1.18	2.09E-33
ENSG00000260766	RP11-226L15.5	-1.17	2.26E-14
ENSG00000174365	SNHG11	-1.17	3.20E-30
ENSG00000267751	AC009005.2	-1.15	7.79E-12
ENSG00000203875	SNHG5	-1.14	2.86E-27
ENSG00000227963	RP5-1074L1.1	-1.14	0.0004053
ENSG00000261664	TTC39A-AS1	-1.12	1.18E-08
ENSG00000260360	RP11-533E19.5	-1.11	0.00029788
ENSG00000255660	RERG-AS1	-1.11	1.96E-10
ENSG00000270681	RP11-372K14.2	-1.11	0.00048987
ENSG00000251580	RP11-539L10.3	-1.10	1.72E-12
ENSG00000245748	RP11-367J11.2	-1.10	0.00013973
ENSG00000228889	UBAC2-AS1	-1.09	6.91E-11
ENSG00000257842	NOVA1-AS1	-1.08	3.28E-07
ENSG00000262823	RP13-580F15.2	-1.07	1.97E-15
ENSG00000228439	TSTD3	-1.07	4.35E-11
ENSG00000258815	RP11-408B11.2	-1.07	2.09E-15
ENSG00000254682	RP11-660L16.2	-1.07	2.70E-14
ENSG00000234352	AC009264.1	-1.07	1.58E-06
ENSG00000257497	RP11-585P4.5	-1.05	0.00015779
ENSG00000263004	RP11-166P13.3	-1.02	2.02E-11
ENSG00000259153	RP6-65G23.3	-1.01	1.97E-13

ENSG00000267302	RP11-178C3.2	-1.00	0.00012796
